# Desensitizing Anxiety Through Imperceptible Change: Feasibility Study on a Paradigm for Single-Session Exposure Therapy for Fear of Public Speaking

**DOI:** 10.2196/52212

**Published:** 2024-07-22

**Authors:** Domna Banakou, Tania Johnston, Alejandro Beacco, Gizem Senel, Mel Slater

**Affiliations:** 1 Event Lab Department of Clinical Psychology and Psychobiology Institute of Neurosciences, University of Barcelona Barcelona Spain; 2 Systems Neuroscience August Pi i Sunyer Biomedical Research Institute Barcelona Spain

**Keywords:** exposure therapy, virtual reality, gradual exposure, fear of public speaking, anxiety, change blindness, public speaking, desensitization, anxiety disorder, feasibility study

## Abstract

**Background:**

Exposure therapy (ET) for anxiety disorders involves introducing the participant to an anxiety-provoking situation over several treatment sessions. Each time, the participant is exposed to a higher anxiety-provoking stimulus; for example, in the case of fear of heights, the participant would successively experience being at a greater height. ET is effective, and its counterpart, virtual reality (VR) exposure therapy (VRET), where VR substitutes real-world exposure, is equally so. However, ET is time-consuming, requiring several sessions.

**Objective:**

This study aimed to compare the results of single-session exposure with those of traditional VRET with regard to reducing public speaking anxiety.

**Methods:**

We introduced a paradigm concerned with public speaking anxiety where the VR exposure occurred in a single session while the participant interacted with a virtual therapist. Over time, the therapist transformed into an entire audience with almost imperceptible changes. We carried out a feasibility study with 45 participants, comparing 3 conditions: single-session exposure (n=16, 36%); conventional multiple-session exposure (n=14, 31%), where the same content was delivered in successive segments over 5 sessions; and a control group (n=15, 33%), who interacted with a single virtual character to talk about everyday matters. A week later, the participants were required to speak on a stage in front of a large audience in VR.

**Results:**

Across most of the series of conventional public speaking anxiety measures, the single-session exposure was at least as effective in reducing anxiety as the multiple-session exposure, and both these conditions were better than the control condition. The 12-item Personal Report of Confidence as a Speaker was used to measure public speaking anxiety levels, where higher values indicated more anxiety. Using a Bayesian model, the posterior probabilities of improvement compared to a high baseline were at least 1.7 times greater for single- and multiple-session exposures compared to the control group. The State Perceived Index of Competence was used as a measure of anticipatory anxiety for speaking on a stage in front of a large audience, where lower values indicated higher anxiety. The probabilities of improvement were just over 4 times greater for single- and multiple-session exposures compared to the control group for a low baseline and 489 (single) and 53 (multiple) times greater for a middle baseline.

**Conclusions:**

Overall, the results of this feasibility study show that for moderate public speaking anxiety, the paradigm of gradual change in a single session is worth following up with further studies with more severe levels of anxiety and a larger sample size, first with a randomized controlled trial with nonpatients and subsequently, if the outcomes follow those that we have found, with a full clinical trial with patients.

## Introduction

Exposure therapy (ET) for anxiety disorders (and other mental health conditions) involves the systematic desensitization over time of patients to feared stimuli or situations. For example, patients with fear of public speaking might be slowly introduced to situations of public speaking: in the first session, perhaps to a photograph of an audience, in the next to a video, and then gradually building toward speaking to a live audience. This has proven over several decades to be an effective therapy [1,2]. Virtual reality (VR) exposure therapy (VRET) uses the same technique, but VR provides the stimuli, relying on the fact that people tend to respond realistically to events in VR [3] and thereby would exhibit anxiety in response to the feared situations. For example, in the case of fear of public speaking, a prerequisite for the success of VRET is that people should exhibit similar anxiety to a virtual audience as they would to a real audience, which has been shown to be the case [4,5]. This approach has been studied and used over the past 3 decades in the treatment of a variety of phobias and mental health disorders, including but not limited to social anxiety disorders, claustrophobia [6], panic attacks, posttraumatic stress disorder, eating disorders [7], and generalized anxiety disorders [8,9]. Early examples can be found in Rothbaum et al [10-12] and in Difede and Hoffman [13], with a review of this early work in Krijn et al [14] and a meta-analysis in Powers and Emmelkamp [15], which showed a large effect size for VRET compared to control conditions and no disadvantage compared to in vivo treatments. This was seen again in a randomized controlled trial (RCT) that specifically considered social anxiety disorders [16]. Moreover, VRET has practical and logistic advantages since the entire treatment can take place in the office of the clinician, rather than arranging for outside visits for the patients to be exposed to real-life events (eg, visiting a high floor of a building in the case of fear of heights or being exposed to a live audience in the case of public speaking anxiety). Moreover, there is evidence suggesting that VRET may be preferred to in vivo therapy [17] by patients with anxiety disorders. Recent meta-analyses have continued to show that VRET leads to therapeutic outcomes that are at least as successful as in vivo treatments [18,19], including cases of severe anxiety, obsessive compulsive, and posttraumatic stress disorders [20] and specifically in relation to public speaking anxiety [21].

VRET has been extensively researched in the treatment of public speaking anxiety [16,22-25], where individuals initially experience anxiety, leading to behavioral, cognitive, and physiological symptoms, when delivering or anticipating the delivery of a speech in front of an audience. ET for public speaking anxiety involves gradual exposure of individuals over various sessions to situations of public speaking, where at each successive session, the situation becomes closer to speaking to a live audience. VRET follows the same idea but with virtual audiences. VR, in particular, offers greater flexibility at low cost in the sense that aspects of the stimuli can easily be changed: audiences can be of different types and be reactive [5], and parameters such as the size of the audience can be changed [16,26,27], including their appearance and behavior [5,28], as well as the virtual location and context of the speech (eg, classrooms, lecture rooms, conference rooms) [24] and the representation of the self [29,30].

ET, whether in vivo or in VR, can be accompanied by cognitive therapy, where the clinician attempts to help the patient reframe their anxious thoughts about the situation. For example, Freeman et al [31] used a simple form of cognitive behavioral ET with VR, where over 5 sessions, patients with fear of heights learned to overcome their fear. Their task was to move to various levels of a building accompanied by a virtual therapist and at each level to perform some tasks on a balcony overlooking an atrium below. Ultimately, they made it to the top floor, and their level of anxiety was significantly reduced compared to before the exposures and to the control condition.

A meta-analysis by Lim et al [32] found an average of 6 VR sessions, each lasting around 37 minutes, among effective VRET interventions. Another meta-study by Hinojo-Lucena et al [33] found that up to 12 sessions are necessary, spread over a week for effective treatment. This is similar to the finding of Reeves et al [21], who reported between 5 and 12 sessions in the papers studied to be effective, while Chesham et al [18] found up to 14 sessions to be effective. Although not in the context of an ET intervention, Boetje and van Ginkel [34] argued that the optimum number of VR sessions for reducing public speaking anxiety cannot be prescribed, since the characteristics of VR treatments vary among the reviewed studies. Following from this, Lim et al [32] argued that the number and length of sessions should be considered as a function of severity of the patient’s condition.

Here, we introduced a new paradigm based on the idea of VRET but with a single VR session during which participants advance toward a situation that would cause greater anxiety, with changes occurring almost imperceptibly. Participants with fear of public speaking are engaged in conversation by a virtual counselor, who explains the issues behind fear of public speaking and encourages the participants to speak about their own public speaking problems and gives them various exercises to do. The counselor is represented by a virtual human character, who stands in front of the person, facing them, and talks and gestures in a natural way. After a while, a copy of the counselor emerges from behind their virtual body, so there are 2 identical instances of the counselor, although only the original one continues the dialogue, while the other one moves off to the side and continues listening. As time progresses, this division process continues, where the additional copies emerge. In addition, after a while, the new copies gradually transform into different virtual human characters. This process continues mainly in peripheral vision, and the changes are imperceptible. Over time, the standing virtual characters adopt a seated position. By the end of the session, the participants find themselves speaking in front of an entire seated audience.

The idea behind why this approach may be effective is to consider at which point the anxiety provoked by public speaking would become active. First, the participant speaks to only 1 (virtual) person. Then, the number of people becomes 2, but it is the same person—the counselor. However, 2 is not an audience, nor is 3. If 3 people are not an audience, are 4? If 4 people are all right, does 1 more matter? Generally, if the participant is comfortable speaking to n people, then imperceptibly making the audience n+1 should make no difference (for n>1). In addition, the changes take place slowly (except for the first), so there is no obvious moment when the participant is not speaking to an audience and then is speaking to an audience. The hypothesis is that people can learn through this process that just as it is possible to speak to 1 person without anxiety, the gradual transition to an audience should not generate anxiety. There is no point of discontinuity, where at one moment the participant is suddenly speaking to an audience but a moment ago was not. The hypothesis is that this learning will carry over to subsequent speeches in front of even larger audiences.

We carried out an experiment to explore the utility of this new paradigm. The participants were not patients but people who report some level of fear of public speaking. Hence, this was not a clinical trial but a feasibility study to assess the efficacy of the paradigm. Hence, the specific aim of this paper was to investigate whether the gradual change paradigm might produce a reduction in the fear of public speaking that is at least as good as the traditional multiple-session VRET approach.

## Methods

### Ethical Considerations

The experiment was approved by the Comissió Bioètica of the University of Barcelona (IRB00003099), and participants provided written informed consent. All methods were performed in accordance with relevant guidelines and regulations.

### Experimental Design

**Figure 1:**
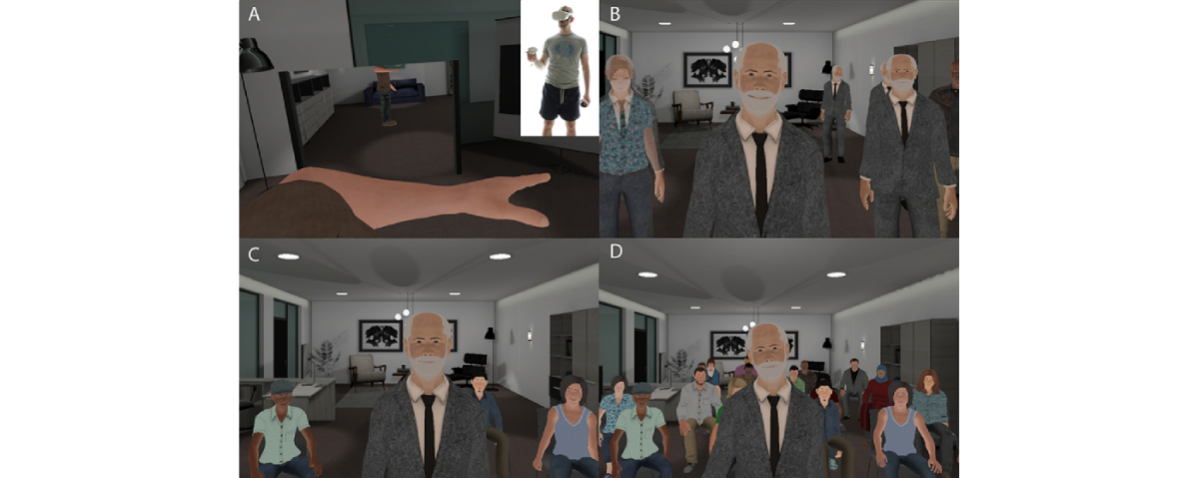
The scenario. (A) A male participant embodied in a male virtual body with a virtual mirror to his left. The inset shows the person with the HMD and controllers. (B) Single-session exposure: the virtual counselor talks to the participant, while new copies of him divide and gradually transform into different virtual human characters. (C) Multiple-session exposure (session 2): the participant is exposed to 3 virtual characters forming the audience. (D) The full audience in both single- and multiple-session conditions. HMD: head-mounted display.

**Figure 2:**
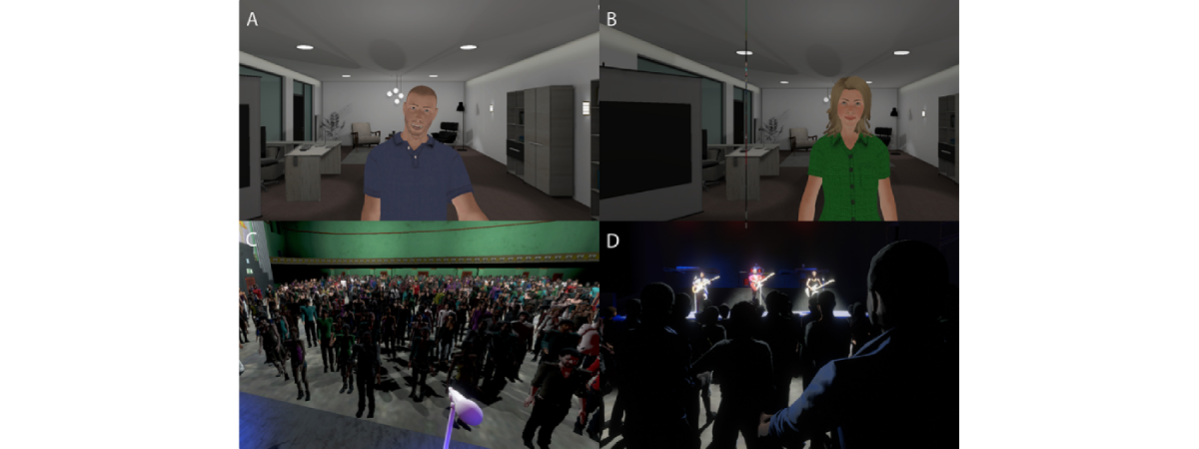
Control condition and concert scenario. (A and B) Virtual humans for the control condition. (C) Virtual audience seen from the stage from the viewpoint of the participant in front of the microphone prior to introducing the band Dire Straits. (D) View from the audience once the concert started.

We conducted the experiment with 45 participants using a between-groups design, with a single factor (exposure) with 3 levels: single-session exposure (n=16, 36%), multiple-session exposure (n=14, 31%), and control group (n=15, 33%). In the single-session exposure, participants experienced the scenario as described in the *Introduction* section—where 1 counselor eventually morphed into an entire audience. The multiple-session exposure group experienced traditional VRET, with 5 sessions, each with an increasing audience size over approximately 3 weeks, with 2 sessions per week (Figure 1). The virtual environment, virtual counselor, and conversation were identical in these 2 groups. Additionally, there was a control group, where participants conversed with a gender-matched virtual human who asked general questions of the participants, such as their name, job, skills, and interests (hobbies), and also talked about themselves (Figure 2A,B). The purpose of the control group was to check that simple exposure to any VR where the participant was required to talk would also be sufficient to reduce anxiety.

At the end of the experiment (single session, multiple sessions after the end of the fifth session, control group), participants were asked to return 1 week later, when they would be on a stage in front of a large audience in a theatre in VR and would be required to introduce a performance by the band Dire Straits (Figure 2C,D). This final exposure was for testing. A historic (1980s) band was chosen so that participants would need to conduct some research to find out more about it. Moreover, since the presentation in front of a large audience was to be several days later, we could assess anticipatory anxiety.

The experimental scenarios are illustrated in Multimedia Appendix 1, which shows a movie of the various conditions.

### Recruitment

Participants were recruited via advertisements around the campus of the University of Barcelona and through social media. Participants had a moderate level of public speaking anxiety. During recruitment, potential candidates underwent a screening process using the 12-item Personal Report of Confidence as a Speaker (PRCS-12) [35], a standard instrument for assessing public speaking anxiety where greater values indicate more anxiety, and the Liebowitz Social Anxiety Scale (LSAS) [36] to identify those eligible to participate (see Multimedia Appendix 2, which also includes details of inclusion and exclusion criteria).

### Materials

We used a wide field-of-view stereo head-tracked, head-mounted display (HMD), through which the participants were embodied in a life-sized (gender matched) virtual body from a first-person perspective, spatially coincident with, and visually substituting their real body. Through upper body tracking, the virtual body moved synchronously with their own body movements. They saw a reflection of the virtual body from the neck down in a virtual mirror and when directly looking toward themselves. Full details of hardware and programming implementation are given in Multimedia Appendix 3, which also contains the scripts of the dialogues between virtual counselors and participants.

### Response Variables

There were 2 types of response variables. First, after the VR exposures, a questionnaire (“VR Questionnaire”) on the level of body ownership and agency over the virtual body and general responses to the virtual audience was administered. The questions are shown in Multimedia Appendix 4; open-ended questions that were used for sentiment analysis were also included. Second, we included the response variables directly related to public speaking anxiety, as shown in Table 1. The “pre” prefix refers to administration of the questionnaire prior to VR exposure, “post” means after the VR exposure and just before the VR concert exposure, and “after” refers to after the concert. Further details of these are given in Multimedia Appendix 5 [37-40].

**Table 1:** Response variables.

Variable	Interpretation	Study
preIAT^a^, postIAT	IAT for fear of negative evaluation Range: –2 to 2 Negative scores: automatic preference for self/rejected, other/liked Positive scores: automatic preference for other/rejected, self/liked	[37]
prePRCA24^b^, postPRCA24	Communication apprehension: level of anxiety triggered by real or anticipated communication Scores: 24-120 (higher scores indicating more anxiety)	[38]
preSPIC^c^, postSPIC, afterSPIC	SPIC Scores: 15-105 (higher scores indicating feeling better about self and considered as more competent performance)	[39]
postSTAI^d^	Short-form STAI administered before the concert speech Scores: 8-32 (higher scores indicating more comfort)	[40,41]

^a^IAT: Implicit Association Test.

^b^PRCA: Personal Report of Communication Apprehension.

^c^SPIC: State Perceived Index of Competence.

^d^STAI: State-Trait Anxiety Inventory.

### Procedures

During the first visit of participants to the VR laboratory, they were given an information sheet to read, and after they agreed to continue with the experiment, they were given a consent form to sign. Next, they completed a series of questionnaires assessing their fear of public speaking and negative evaluation (*prePRCA24*, *preSPIC*, *preIAT*, and *preSTAI*, where PRCA refers to Personal Report of Communication Apprehension, SPIC refers to State Perceived Index of Competence, IAT refers to Implicit Association Test, and STAI refers to State-Trait Anxiety Inventory) that also served as baseline measures, as well as other demographic data (see Multimedia Appendix 6 for details). Before the experiment started, participants were fitted with a head-mounted display (HMD) and body-tracking equipment. The view seen through the HMD was calibrated for each person. Participants in the multiple-session exposure group returned approximately 2 days later for the second session and so on until they completed all 5 treatment sessions. After the last VR exposure, participants completed a post-VR experience questionnaire. One week after participants’ single (single-session or control exposure group) or final exposure (multiple-session exposure group), they returned for a follow-up session, where they had to give the Dire Straits welcome speech. Before delivering the speech, they completed the questionnaires assessing their fear of public speaking and negative evaluation again (*postIAT*, *postPRCA24*, *postSPIC*, *postSTAI*). When they finished and came out of the virtual environment, they completed a post-VR questionnaire and a questionnaire on the perceived index of competence (*afterSPIC*) related to the speech they had just delivered. Next, they were debriefed about the purpose of the study and compensated, and they left the laboratory.

### Statistical Analysis

We did not make statistical inferences about the questionnaire variables related to the VR experience (eg, body ownership; see Multimedia Appendix 4) but only wished to check that the results conformed with earlier studies. Hence, we only considered these response variables at the descriptive level.

For variables specifically related to anxiety, we used a Bayesian statistical model detailed later, equivalent to 1-way ANOVA, for all but 1 response variable and a logistic model for postIAT. This resulted in posterior distributions for each of the parameters of the model, from which we could compute any probabilities of interest. The model included all response variables simultaneously, so there was no issue with multiple comparisons that would result in problems in the interpretation of significance levels in classical null hypothesis testing.

For each response variable (postIAT, postPRCA24, postSPIC, afterSPIC), the linear predictor, which related the independent variables to the mean of the response variables, was of the form

Condition + Covariate + (Covariate × Condition) [+ familiarity] **(1)**

where *Condition* refers to the main effects of control, multiple-session, and single-session conditions; *Covariate* refers to the *pre* variables (Table 1); *Covariate* × *Condition* is the interaction effect; and the covariate *familiarity* refers to the scores on a question about the familiarity of participants with the band Dire Straits. Since the SPIC variables and postSTAI related to preparation for giving a talk about Dire Straits, we also included familiarity for these response variables as a covariate based on the results of Figure S4 in Multimedia Appendix 7, which shows a lower level of familiarity for the multiple-session condition compared with the other conditions. There was no *pre* covariate for postSTAI.

The linear predictor for each response variable was therefore of the form:



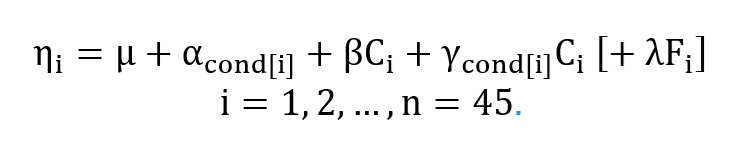



**(2)**

This is a standard ANOVA model where:

- μ is the grand mean;

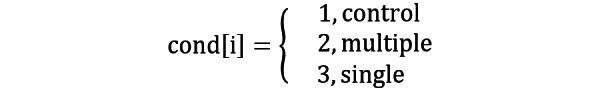

- α_j_ is the main effect for condition;
- γ_j_ is the interaction between the condition and the covariate, where j=1 (control), 2 (multiple sessions), or 3 (single session), with α_1_=γ_1_=0 so that multiple-session and single-session conditions were compared against the control condition;
- C_i_ is the *pre* variable as a covariate (eg, preIAT); and
- F_i_ is familiarity (used for postSPIC, afterSPIC, and postSTAI).

Since in the case of postSTAI, there was no *pre* covariate, the corresponding model was reduced to the main effect together with F_i_.

In the case of all response variables, except for postIAT, we used an ANOVA model with a covariate, so the mean (μ_i_) of the response variable (y_i_) was set equal to the linear predictor:

μ_i_=η_i_

**(3)**

In standard ANOVA, the likelihood (ie, the distribution of the response variable conditional on the parameters) is required to have a normal distribution. Here, we can be more flexible and let the likelihood follow a Student *t* distribution. This has the advantage that it has a wider dispersion than the normal, thus allowing for potential outliers. In addition, for high enough degrees of freedom, it approximates the normal, and for degrees of freedom of about >30, it is indistinguishable. Hence, the likelihood is:

y_i_ ~ Student_t(υ, μ_i_, σ)

**(4)**

where υ>1 is the degrees of freedom parameters, μ_i_ is the mean, and σ is the scale parameter. Smaller values of υ and larger values of σ correspond to greater dispersion with respect to the symmetric distributions around μ_i_. For larger υ values (>30 approximately), the distribution is equal to the normal distribution, with mean μ_i_ and SD σ.

In the case of postIAT, this model did not produce a good fit to the data. This is because IAT is bounded by the values 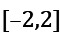 by construction. Hence, we normalized the postIAT and preIAT values by transforming them to a 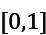 scale. Next, we used the Beta distribution for the likelihood: Beta was chosen because the probability density is bound to 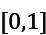, and the distribution can take on many different shapes (symmetric about 0.5, J shaped, inverse J shaped, uniform, etc) and therefore can adapt to the data. Hence, in the case of IAT, the model is:

y_i_ ~ Beta(ϕμ_i_, ϕ[1 – μ_i_])

**(5)**

where ϕ>0 is a scaling parameter, and the mean of the distribution is μ_i_. To ensure that
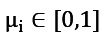
, we used the logit link function between the mean and the linear predictor, with the inverse:



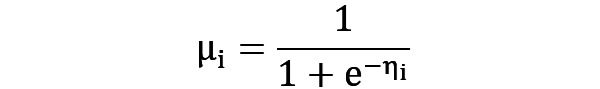



**(6)**

This is a standard logistic model.

We used weakly informative prior distributions (ie, proper probability distributions with wide variance [42,43]). All β_j_ ~ normal(mean 0, SD 100); hence, the prior 95% credible intervals (CIs) were –200 to 200. All σ, υ, ϕ ~ Gamma(2,0.1); hence, the 95% prior CIs were 2.4-55.7.

All response variables were in the same overall model, so the parameters were, for example, denoted by μ_prc_, α_prc_, β_prc_, γ_prc_, υ_prc_, and σ_prc_ in the case of postPRCA24, and the others are shown in Table S4 in Multimedia Appendix 8.

The analysis was carried out using the probabilistic programming language Stan [44] with the rstan interface in R (R Foundation for Statistical Computing) [45]. In total, 3000 iterations were used with 4 chains, and all simulations converged successfully, with all Rhat=1, indicating that the results of the chains were properly mixed.

## Results

### Participant Statistics

The mean age of the 45 participants was 26.1 (SD 7.36) years, 27 (60%) of them identified as female, 14 (31%) as male, and the remaining 4 (9%) as “other” or “preferred not to say.” The detailed demographics are provided in Multimedia Appendix 6.

### Descriptive Statistics for VR Questionnaire Results

The median levels of body ownership and agency were high and in line with the results of previous experimental studies (eg, [46]), although body ownership and agency were of peripheral interest in this study, and the participants only saw their virtual body in a mirror for a short period prior to the discussion with the virtual human and prior to introducing the band Dire Straits in the second phase. Positive responses to the audience were middle to high in the single- and multiple-session conditions but somewhat lower in the control condition. Full details and analysis of these responses are available in Multimedia Appendix 7.

### Descriptive Statistics for Anxiety-Related Results

Table 2 shows the means and SEs of the response variables that were directly related to public speaking from Table 1. It also shows the effect sizes (Cohen d) for the difference between *post* and *pre* measures and the effect sizes for the *post-pre* differences comparing between conditions. The *post-pre* effect sizes for the control group were all small, except for afterSPIC, which had a medium effect size. Comparing the single- and multiple-session conditions, the *post-pre* effect sizes for the single-session condition were always at least as strong as those for the multiple-session condition. In addition, the change in IAT showed a medium effect size for both conditions, a medium effect size for afterSPIC in the single-session condition, and a small effect size for the others. Since postSTAI was only measured just before the concert speech, there was no *post-pre* effect size. Most of the effect sizes for comparison between the conditions were small to medium, except for the difference between the single-session exposure and control conditions, where the effect size was large for afterSPIC.

The effect sizes are crude overall measures and do not consider the relationship between *pre* and *post* measures, nor do they include the covariate familiarity (how much participants were familiar with the band Dire Straits). We now consider the statistical model that included covariates.

**Table 2:** Means (SEs) of response variables by condition, paired effect sizes (Cohen d) for differences between post and pre measures, and effect sizes (Cohen d) for post-pre values comparing between conditions.

Condition		IAT^a^	PRCA24^b^	postSPIC^c^	afterSPIC	postSTAI^d^
**Single-session exposure**						
	Mean (SE)	0.35 (0.15)	5.38 (2.49)	–2.31 (3.00)	5.88 (2.94)	23.2 (1.27)
	Cohen d *post*-*pre*	0.62	0.26	–0.21	0.58	—^e^
**Multiple-session exposure**						
	Mean (SE)	0.30 (0.17)	0.00 (3.36)	2.29 (4.77)	–5.36 (6.84)	20.7 (0.93)
	Cohen d *post*-*pre*	0.59	0.00	0.16	–0.32	—
**Control**						
	Mean (SE)	–0.10 (0.20)	4.93 (2.50)	–7.20 (2.31)	–11.73 (3.35)	20.6 (1.28)
	Cohen d *post-pre*	–0.18	0.24	–0.35	–0.50	—
Cohen d *post-pre* (single- vs multiple-session exposure)		0.075	0.478	–0.306	0.577	0.539
Cohen d *post-pre* (single-session exposure vs control group)		0.639	0.045	0.459	1.423	0.489
Cohen d *post-pre* (multiple-session exposure vs control group)		0.564	–0.441	0.680	0.317	0.026

^a^IAT: Implicit Association Test.

^b^PRCA: Personal Report of Communication Apprehension.

^c^SPIC: State Perceived Index of Competence.

^d^STAI: State-Trait Anxiety Inventory.

^e^Not applicable.

### Statistical Analysis of Anxiety-Related Results

Summaries of all the posterior distributions of the model’s parameters are given in Multimedia Appendix 8, with further details of the results including the model’s goodness of fit and the availability of data and programs for analysis.

The main concern of interest was to check whether the single-session condition is at least as effective in contributing to a diminution of anxiety compared to the multiple-session condition and whether these are different from the control group. We considered each response variable in turn, noting that all CIs were substantially narrower than their priors.

#### Implicit Association Test

The interaction terms for multiple- and single-session conditions had CIs mainly in the positive region with high (single session, 0.945) and moderately high (multiple sessions, 0.817) probabilities of being positive. In other words, greater values of preIAT were associated with greater levels of postIAT. Moreover, the 2 CIs were similar in their range. The coefficient of preIAT for the control group showed no evidence of being different from 0 (probability of being positive=0.458). These results point to there being no or little difference between the multiple- and single-session exposures with respect to their effect on IAT, and each of these were different from the control group. Hence, postIAT increased with preIAT for the multiple- and single-session groups. However, for the single-session group, the probability that the rate of increase (slope) was greater than 1 was 0.835, whereas it was 0.601 for the multiple-session group.

#### Personal Report of Communication Apprehension

In the control group, there was clearly a positive slope between postPRCA24 and prePRCA24 (probability=1.000, β_prc_; see Table S4 in Multimedia Appendix 8). This slope was reduced in the case of both multiple sessions (probability = 1 – 0.023 = 0.977) and the single session (probability = 1 – 0.077 = 0.923). The CIs for the interaction terms for the multiple- and single-session groups were similar. Although both multiple- and single-session exposure might have reduced the slopes of postPRCA24 on prePRCA24, there was no important difference between them, and they were both different from the control group. Hence, both single- and multiple-session interventions reduce the proportional increase in postPRCA24 with respect to prePRCA24.

#### The postSPIC Variable

This was administered prior to the participants presenting the Dire Straits welcome speech in front of a virtual audience. In the control group, postSPIC was positively linearly related to preSPIC (probability=1.000, coefficient was positive). In the multiple-session group, there was a high main effect (mean 47.78, probability=1.000), but the slope on preSPIC was reduced compared to the control group by –0.67 (probability of being negative = 1 – 0.002 = 0.998). The single-session condition also had a high main effect of 26.29 (probability of being positive=0.895), and the slope on preSPIC was reduced by a mean of –0.33 (probability of being negative = 1 – 0.142 = 0.858). The familiarity variable was positively associated with postSPIC (probability=0.810), consistent with the likelihood that the presentation task would be less stressful for those with prior knowledge of Dire Straits.

#### The afterSPIC Variable

This was administered after the presentation of the Dire Straits welcome speech to the audience. As before, the control condition was positively associated with preSPIC (probability=1.000). The multiple-session condition had a strong positive main effect of 68.06 (probability of being positive=0.999) but with a reduction in the slope on preSPIC by –1.02 (probability of being negative = 1 – 0.001 = 0.999). The single-session condition also had a strong positive main effect of 51.42 (probability=0.968) but decreased in slope on preSPIC by –0.52 (probability = 1 – 0.096 = 0.904). There was a high probability (0.970) that familiarity with Dire Straits was positively associated with afterSPIC. For both postSPIC and afterSPIC, there was a difference for both multiple- and single-session exposure compared with the control group.

#### The postSTAI Variable

The single- and multiple-session conditions had similar posterior distributions, and their main effects were greater than those of the control condition. postSTAI was positively associated with familiarity.

**Figure 3:**
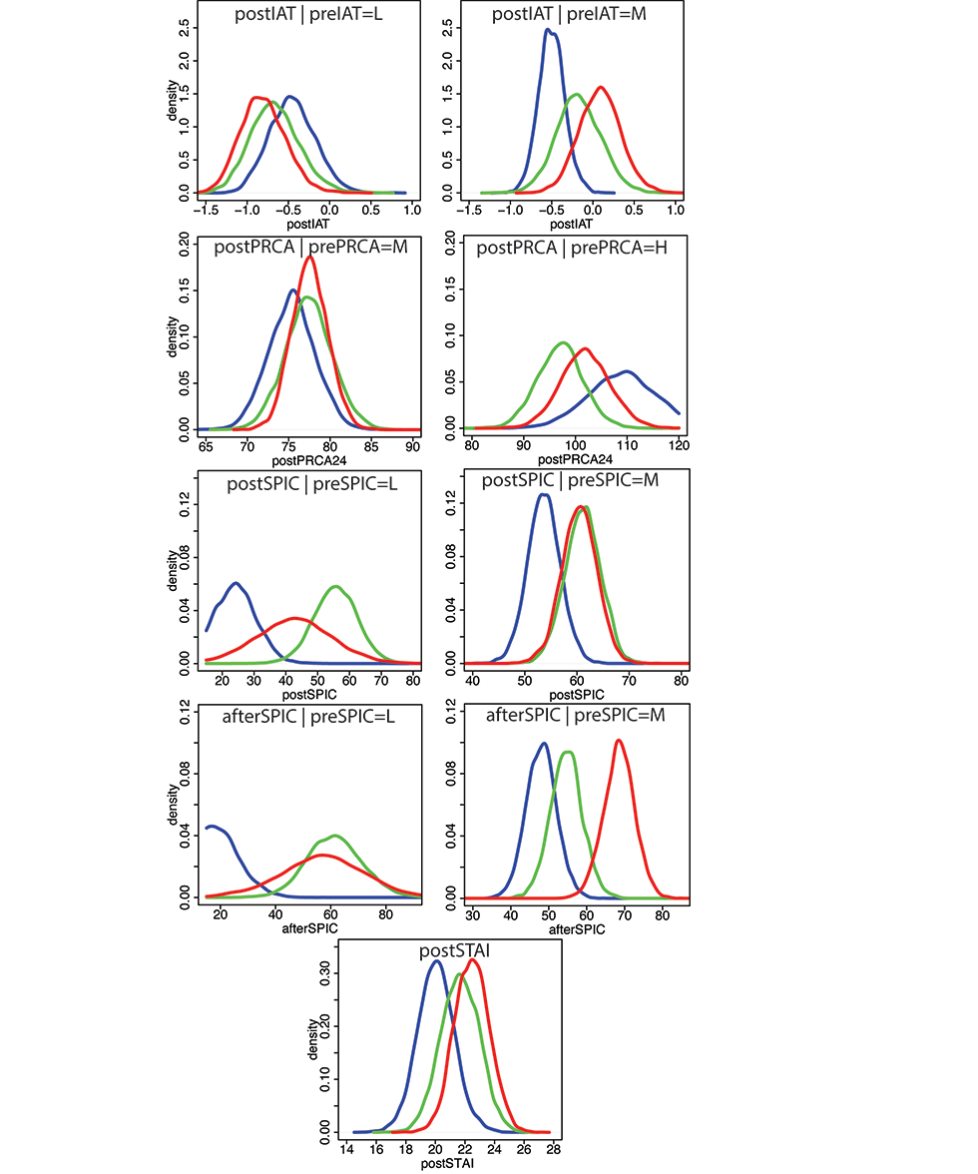
Posterior distributions for each response variable using equation 2. The red curves are for the single-session condition, green for the multiple-session condition, and blue for the control condition. For all except postSTAI, these are conditional distributions with the prevariable: lowest (L), middle (M), or highest (H) values. For postSTAI, it is the unconditional distribution. The familiarity variable is set at its median of 3. IAT: Implicit Association Test; PRCA: Personal Report of Communication Apprehension; SPIC: State Perceived Index of Competence; STAI: State-Trait Anxiety Inventory.

From the statistical model, the posterior distributions of each response variable can be obtained for any values of the covariates and the familiarity variable using equation 2. Figure 3 shows all the posterior distributions conditional on the covariates being 10% greater than the lowest-possible value, the middle value, and 10% lower than the highest-possible value. Hence, if the range of the variable is xmin-xmax and if d = xmax – xmin, then these values are xmin + 0.1d, (xmin + xmax)/2, and xmax – 0.1d, respectively. From Figure 3, we can see that the control distributions were clearly different from those of the multiple- and single-session conditions. Multiple sessions gave better results than the single session only in the case of postPRCA24, conditional on prePRCA24 being at the highest level, as defined earlier, but in this case, both multiple- and single-session conditions were superior in their effects than the control condition. For postIAT, postSPIC, afterSPIC, and postSTAI, the single-session condition was always at least as good as the multiple-session condition.

Table 3 shows probabilities computed from the distributions in Figure 3, particularly the probabilities of improvement. For all variables except *postPRCA24*, improvement corresponded to an increase in the *post* score compared to the *pre* score. For postPRCA24, improvement corresponded to a decrease in scores. For postSTAI, there was no *pre* score. Formally, the probabilities are as follows:

P(post > x | pre = x, observed data) 

**(7)**

where x is the lowest and middle of all variables except postPRCA24.

For postPRCA24, the probabilities are as follows:

P(post < x | pre = x, observed data)

**(8)**

where x is the middle and highest. In the case of postSTAI, there was no conditioning on *pre*=x.

For PRCA24, since lower values indicated better outcomes with respect to public speaking anxiety, we were only interested in the probability that postPRCA24 (after exposure) was less than that when prePRCA24 was equal to the middle and highest scores before exposure (ie, these indicate improvement). For all other variables, higher values represented better outcomes, so we were interested in the probability that the *post* values were greater than the *pre* values for the lowest and middle *pre* settings. Probability ~ lowest means probability > lowest or probability < lowest, depending on whether the symbol above the column of probabilities is > or <. For example, the probability that postPRCA24<110.4 conditional on prePRCA24=110.4 was 0.958 in the single-session condition. For postSTAI, there were no *pre* values, so the probability that postSTAI>0 was 0.510 in the control condition and 0.900 in the multiple-session condition. Where relevant in equation 2, the familiarity variable was set at its median value 3.

From Table 3, we can see that the control condition had the least probability of improvement in every case but 2 (lowest IAT and middle PRCA24). Generally, the single-session condition was only notably worse than the multiple-session condition in the case of the middle postPRCA24, but in this case, the probabilities were in any case low. For afterSPIC, the single-session condition had a probability of improvement more than 9 times greater than that of the multiple-session condition for the middle score.

We also considered the odds of improvement (ie, ratios of probabilities). In the case of postIAT, with the preIAT at the middle setting, the odds of improvement were 0.257/0.001 = 257 for the multiple-session condition compared to the control condition and 0.631/0.257 = 2.1 for the single-session condition compared to the multiple-session condition. For postPRCS24, with the highest prePRCS24, the odds of improvement for the multiple- and single-session conditions over the control condition were at least 1.7 (0.958/0.577). For postSPIC, the odds of improvement with the middle preSPIC were 27 for the multiple-session condition and 24 for the single-session condition over the control condition. For lowest afterSPIC, the multiple- and single-session conditions had odds of improvement of 4.2 and 4.3, respectively, and in the case of middle afterSPIC, the multiple-session condition had odds of improvement of 53 over the control condition, while the single-session condition had odds of improvement of 9 over the multiple-session condition. For middle postSTAI, the odds of improvement were 1.8 and 1.9 for the multiple- and single-session conditions, respectively, over the control condition.

**Table 3:** Probabilities of response variables showing an improvement in the 3 conditions.

Response variable and conditions			Lowest^a^		Middle^a^		Highest^a^		Probability ~ ^b^ lowest		Probability ~ middle		Probability ~ highest
**postIAT^c^**			–1.6		0		1.6		>		>		—^d^
	Control							1.000		0.001			
	Multiple sessions							1.000		0.257			
	Single session							1.000		0.631			
**postPRCA24^e^**			33.6		72.0		110.4		—		<		<
	Control									0.116		0.577	
	Multiple sessions									0.029		0.998	
	Single session									0.006		0.958	
**postSPIC^f^**			24		60		96		>		>		—
	Control							0.006		0.023			
	Multiple sessions							0.000		0.632			
	Single session							0.026		0.556			
**afterSPIC**			24		60		96		>		>		—
	Control							0.232		0.002			
	Multiple sessions							1.000		0.107			
	Single session							0.980		0.978			
**postSTAI^g^**			10.4		20		29.6		>		>		—
	Control							1.000		0.510			
	Multiple sessions							1.000		0.900			
	Single session							1.000		0.982			

^a^The values for *lowest*, *middle*, and *highest* are for the *pre* variates.

^b^The symbol ~ stands for > or <, depending on the variable concerned.

^c^IAT: Implicit Association Test.

^d^Not applicable.

^e^PRCA: Personal Report of Communication Apprehension.

^f^SPIC: State Perceived Index of Competence.

^g^STAI: State-Trait Anxiety Inventory.

### Sentiment Analysis of Participant Comments

As mentioned earlier, participants were asked open-ended questions for comments on their experience at the end of the first phase of the study (the treatment: single session, multiple sessions, or control) and again after the end of the second phase (the test: introduction of the concert 1 week later). Their responses were used to carry out sentiment analyses to examine the extent to which sentiment varied across the experimental conditions. This is described in detail in Multimedia Appendix 9. Sentiment scores for both the treatment and test comments fell into 2 distinct low- and high-sentiment clusters. Those in the single- and multiple-session groups were more than twice as likely to be in the high-sentiment cluster than the low-sentiment one, whereas those in the control group were approximately the same in both. For the test condition, similar results were found, except that only those in the single-session group had more than double the likelihood to express high compared to low sentiment, whereas those in the control group were doubly likely to express low sentiment. It is noteworthy that those in the control group typically indicated the nervousness they felt during the period before they introduced the band. With the relatively low sample size, we cannot conclude that these findings would generalize to the population of people with fear of public speaking, but they are compatible with what might be expected.

## Discussion

### Principal Findings

ET typically requires multiple sessions, with patients being gradually exposed to increasing levels of anxiety-producing stimuli. Here, we introduced a new paradigm based on gradual change within a single session. The participant begins by talking to a single counselor standing in front of them, but by the end of the session, that single counselor morphs into a seated audience. We elicited response variables prior to VRET and then both before and after participants introduced a music band in front of a large virtual theater audience. The single-session condition was compared with traditional VRET of 5 sessions and with a control group. The results indicate that single-session exposure results in outcomes that are at least as favorable as those in multiple-session exposure on most measures and that both single- and multiple-session conditions result in outcomes that are typically better than those of the control condition.

### Comparison With Prior Work

The results, as represented by the effect sizes (Table 2), were mostly moderate. There was a clear distinction between the control group and the treatment groups, and the *post-pre* effect sizes ranged from 0 to 0.62 (considering absolute values). However, our results compare well with those of a meta-analysis reported by Chesham et al [18], where across the 9 studies using VRET for social anxiety that met their inclusion criteria and the 13 sets of results within those studies, the *post-pre* effect sizes (Hedge g) ranged from –0.56 to 1.57 (median 0.486, IQR 0.054-0.604). Considering 6 studies that compared VRET with a waiting group, the overall effect size was 0.82. Similarly, a meta-analysis of 13 papers on social and performance anxiety was reported by Carl et al [19]. Overall, the median effect size (Hedge g) was 0.61 (IQR 0.14-0.91). Note that Hedge g and Cohen d are similar. For comparison only with a waiting-list group, the median effect size was 0.91 (IQR 0.68-1.33), and for comparison in vivo, the median was 0.61 (IQR 0.14-0.91). A recent meta-analysis [32] found similar (in fact, slightly lower) effect sizes across 92 studies on public speaking anxiety. A further recent meta-analysis was reported by Reeves et al [21] with higher effect sizes, although breakdowns across individual studies and measures were not presented. As a further example, an RCT [23] included 3 groups (cognitive behavioral therapy [CBT], cognitive behavioral therapy in virtual reality [VRCBT], and waiting list) and 12 sessions of ET. There was no difference between the CBT and VRCBT groups, so they were combined into 1 treatment group. Across a range of variables, comparisons of the waiting-list group with the treatment group found effect sizes for anxiety reduction in the range of 0.58-1.00. Good results were maintained at 1-year follow-up [47]. In a study comparing in vivo ET (6 sessions) with VRET (4 sessions) and a waiting list [16], effect sizes (Cohen d) were compared for the VR group and the waiting-list group (posttreatment controlling for pretreatment) across a number of measures. When comparing the VR group with the waiting-list group, the median effect size was 0.74 (IQR 0.63-0.93), and when comparing in vivo with the waiting-list group, the median effect size was 0.72 (IQR 0.48-1.00). At 12-month follow-up, the maximum effect size was 0.61. The effect sizes for all these studies were across a range of different instruments. In general, evidence suggests that results from VRET do generalize to real life [48].

The effect sizes found in our study are in line with findings in the literature. However, the effect size is a blunt instrument dealing only with overall average effects. We propose that the type of analysis illustrated in Figure 3 gives richer information. Here, we can immediately see the differential effects of the treatments, depending on the starting state of the participants. For example, if the IAT is low to start with (greater self-rejection), all the conditions lead to an improvement. However, at the medium level of the initial IAT, there is a differential effect of the 3 methods, with single-session exposure resulting in a greater probability of higher IAT scores and the control condition being clearly ineffective. This can be seen even more clearly in the case of both postSPIC (anticipatory anxiety prior to the concert announcement) and afterSPIC (after the concert announcement).

The idea of single-session ET has been discussed before, although with a quite different paradigm. A 1-session VRET for public speaking anxiety was introduced by Lindner et al [49], with strong positive results compared to a waiting-list group. However, this involved an extensive 3-hour intervention, including a VR session, followed also by a 4-week in vivo transition program. The paradigm was similar to an earlier one for treatment of spider phobia, which had similar positive results [50].

ET is thought to be effective due to either habituation through systematic desensitization or, alternatively, classical extinction [1,51-53]. In the latter case, the stimulus (an audience) is presented multiple times but under conditions where the normal response is avoided through inhibitory control. It is unlikely that the new paradigm presented here can be explained through either habituation or extinction. Although there is exposure, it is implicit rather than explicit (ie, the focus of the participant is on the discussion with the single individual throughout). Moreover, the situation, at least to start with, is a bizarre one, with multiple copies of the same individual appearing but taking no part in the proceedings. We postulate that the positive effect of the treatment occurs through generalization from speaking to a single individual: the participant implicitly learns that speaking to an audience is not different from speaking to an individual; if the latter is possible without anxiety, then so is the former.

The model of social anxiety disorder proposed by Clark and Wells [54] is useful in understanding the outcome. Although the model applies to the more general situation, public speaking anxiety is a specific case of social anxiety. In particular, some of the elements that contribute to instances of social anxiety are relevant to the gradual and implicit exposure paradigm. One component of the model is “perceived social danger,” where individuals predict their own negative performance. In this case, there is a conversation with an individual, and the required performance is carefully guided (eg, read out numbers from 1 to 20 or a piece of text, or describe a movie). “Anticipatory anxiety leading to worry” is where individuals focus on past or imagined failures and thereby predict their own negative behavior. This again is unlikely to occur in the situation where participants are talking to a single individual and, moreover, where the conversation is precisely about public speaking anxiety and where there is counseling about how to prepare for a talk. “Processing of self as a social object” is where individuals’ own negative model of themselves is projected onto others so that they think that others perceive them in this negative way. However, the conversation with the single individual, someone who apparently understands and gives advice about public speaking anxiety, may militate against this. The participant is not in a situation of being evaluated but rather in one where help and advice are available. Overall, we suggest that the encounter with the counselor provides an opportunity for implicit learning that a talk in front of an audience is not fundamentally different from talking with an individual. It is an example of where a single positive experience of talking in front of an audience could generalize to other situations. Using a different method, it was found by Shadinger et al [55] that when college students were instructed to make positive affirmative statements about their forthcoming public speaking performance, this reduced their anticipatory anxiety, suggesting that a single positive experience can generalize.

Nevertheless, although the conversation is with a single individual, an audience does gradually emerge, with changes taking place largely in peripheral vision, with the focus of attention on the counselor. Suppose that some individuals experience a degree of change blindness [56,57] where they were not consciously aware of the growing audience most of the time. In this case, how could it be possible that the audience might have an influence? It has been shown that in change blindness, when participants do not consciously see the changes, these, nevertheless, influence their subsequent decision-making [58]. Even if the audience might hardly be noticed during the time of the conversation, although it is obvious by the end, participants might still be influenced by the fact that they are having a conversation with a growing audience present. Change blindness has been observed in VR [59,60], and our recent study shows that it operates with respect to virtual bodies that are subject to gradual change, even though participants are looking toward them all the time [61].

### Limitations

Although some outcome measures were taken after the introduction of the band Dire Straits at the virtual concert and hence 7 days after the main VR treatment, there is a need for longer-term follow-up for this new paradigm. Here, we were concerned about providing an initial evaluation, and the positive results are encouraging to undertake an RCT with a larger sample and then later a clinical study with longer-term follow-up. The RCT would also include individuals with a higher level of public speaking anxiety.

Consumer VR devices that are entering the market now have built-in eye and facial tracking. Eye tracking especially would be useful to determine how much participants do indeed pay attention to the emerging audience, and eye tracking and facial expression tracking could also provide real-time measures of the extent of ongoing anxiety. Furthermore, the method by which the audience is introduced can be explored. Our approach was based on the idea of maintaining the whole exposure as a conversation with a single individual and emphasizing that by the fact that all new characters emerged from, and were initially clones of, that individual. Moreover, we adopted the method that the copies would gradually transform into other characters and eventually sit down. It is possible that similar results might have been obtained had characters appeared 1 by 1 at different places in the room. Here again, an audience would gradually form but not related to the counselor. Our view is that the connection with the counselor is essential (to maintain the idea that this was a conversation with an individual and not with a group), but this would be interesting to study. Another possibility is that the entire audience might gradually become visible over the course of the conversation, already as different seated characters. Although this is possible, again our view is that the morphing of the counselor into an audience is an essential part of the method.

### Conclusion

Our initial results in testing the feasibility of this paradigm are encouraging and worthy of further research. Although we used the gradual change method in the context of public speaking anxiety, it could also be used for other anxiety states. For example, for fear of heights, the participant could be talking to a virtual counselor, initially at the same level, but imperceptibly the adjacent ground level could move lower and lower until, ultimately, the participant and counsellor are standing near a precipice. With, for example, a phobia of spiders, the participant could interact with a butterfly that gradually morphs into a spider. In the case of agoraphobia, the participant might start talking with a counselor in a closed safe space that gradually morphs into an open shopping area. Further studies of this paradigm are needed in a variety of situations in order to test its efficacy.

## Appendix

Multimedia Appendix 1 Movie illustrating the scenario.

Multimedia Appendix 2 Participant recruitment.

Multimedia Appendix 3 Implementation.

Multimedia Appendix 4 Virtual reality questionnaire.

Multimedia Appendix 5 Response variables.

Multimedia Appendix 6 Participant statistics.

Multimedia Appendix 7 Virtual reality questionnaire responses.

Multimedia Appendix 8 Summary of posterior distributions and goodness of fit.

Multimedia Appendix 9 Sentiment analysis.

## Abbreviations

CBT: cognitive behavioral therapy
ET: exposure therapy
HMD: head-mounted display
IAT: Implicit Association Test
PRCA: Personal Report of Communication Apprehension
PRCS-12: 12-item Personal Report of Confidence as a Speaker
RCT: randomized controlled trial
SPIC: State Perceived Index of Competence
STAI: State-Trait Anxiety Inventory
VR: virtual reality
VRCBT: cognitive behavioral therapy in virtual reality
VRET: virtual reality exposure therapy
