# Supplementary material for: Desensitizing Anxiety Through Imperceptible Change: Feasibility Study on a Paradigm for Single-Session Exposure Therapy for Fear of Public Speaking
Source: JMIR Form Res. 2024 Jul 22;8:e52212. doi: 10.2196/52212 (PMC11301124; doi:10.2196/52212)
Supplement: Multimedia Appendix 2 [file formative_v8i1e52212_app2.docx]

# Multimedia Appendix 2 - Recruitment

## Participants

There were complete data on 45 adult healthy participants with correct or corrected vision who were recruited by advertising around the campus of University of Barcelona and through social media. Most of them had no prior experience of our virtual reality system and were comparable across different parameters, such as knowledge of computers and programming, time they spent playing video games etc. (Multimedia Appendix 6, Table 6.1). Participants were compensated for participation depending on the number of sessions. The amount ranged between €30 and €60 (Euros). Following the completion of the last phase of the experiment, participants were debriefed about the purpose of the study.

## Inclusion Criteria

During recruitment, potential participants underwent a screening process to identify those eligible to participate (Brief Personal Report of Confidence as Speaker - PRCS-12 and Liebowitz Social Anxiety Scale – LSAS. The PRCS-12 has been validated with the Spanish population^[[1]](#footnote-1),^^[[2]](#footnote-2)^ and it was used to guarantee a minimum level of fear of public speaking for candidates to be able to participate in the experiment, with a cut-off of 32 points or more, indicating moderate to high fear of public speaking. Furthermore, the LSAS scale was used to assure that only participants with social anxiety related to fear of public speaking took part in the study, and to avoid recruiting participants with other social phobias that could lead to confounding results. The screening was completed online; the PRCS-12 and LSAS questionnaires were administered using the Qualtrics^[[3]](#footnote-3)^ software. The means and standard deviations of the variables used for inclusion are shown in Table 2.1.

### Table 2.1 – Means and SD of the variables used for inclusion

| Condition | PRCA-12 | LSAS |
| --- | --- | --- |
| Single Exposure | 47.9 ± 8.23 | 42.1 ± 11.15 |
| Multiple Exposures | 46.9 ± 10.02 | 38.3 ± 12.94 |
| Control | 43.1 ± 12.08 | 38.2 ± 15.04 |

## Exclusion criteria

Potential participants currently under psychological treatment or diagnosed with any mental disorder including post-traumatic stress disorder, suffering drugs or alcohol abuse, psychotic symptoms, mental retardation, present substantial visual or hearing deficits, cognitive or organic brain dysfunction, suffering seizure disorders or epilepsy were not eligible to take part in the study.

1. Saura CJI, Carrillo FXM, Montesinos MDH, Alcázar AIR, Amorós MO. Cuestionarios, inventarios y escalas de ansiedad social para adolescentes: una revisión crítica. Revista de psicopatología y psicología clínica. 2003;8(1):1-21. [↑](#footnote-ref-1)
2. Carrillo FXM, Saura CJI, Montesinos MDH. Propiedades psicométricas del Cuestionario de Confianza para Hablar en Público: Estudio con una muestra de alumnos de enseñanzas medias. Psicothema. 1999:65-74. [↑](#footnote-ref-2)
3. <http://www.qualtrics.com/> [↑](#footnote-ref-3)
