# Supplementary material for: Desensitizing Anxiety Through Imperceptible Change: Feasibility Study on a Paradigm for Single-Session Exposure Therapy for Fear of Public Speaking
Source: JMIR Form Res. 2024 Jul 22;8:e52212. doi: 10.2196/52212 (PMC11301124; doi:10.2196/52212)
Supplement: Multimedia Appendix 3 [file formative_v8i1e52212_app3.docx]

# Multimedia Appendix 3 – Materials

## Equipment

The main equipment was an Oculus Rift S head-mounted display and controllers. The head-mounted display has a refresh rate of 80 Hz, and uses a single fast-switch LCD panel with a resolution of 2560 ×1440 pixels, its weight is 555 g. When wearing the headset participants can see the Virtual Reality scenario in 3D and in stereo with a 115º field of view as if they were using their head gaze normally to look around a scene. It has built-in audio that uses speakers embedded in the headset itself, positioned slightly above the participant’s ears. The controllers are used to track the participant’s upper body movements and rotations in real time.

## Implementation

The virtual environment was implemented on the Unity3D^[[1]](#footnote-1)^ platform. The virtual room in which the sessions with the virtual counselor took place was the same for all conditions. It was designed to look like a therapist’s office (Figure 1) populated with furniture and relevant decorative material (e.g., wall paintings and posters). For the Control condition, the decoration was removed. A virtual mirror was located to the left of participants, which helped them inspect their virtual body. The animation-enabled models of the virtual bodies for both the participant and the virtual audience in the concert scenario were created with Adobe Fuse^[[2]](#footnote-2)^ academic version and Mixamo^[[3]](#footnote-3)^. The counselor avatar was the one previously used.^[[4]](#footnote-4)^ The virtual counselor for the two experimental conditions and the generic avatar for the control condition, as well as the audience were all located in the same places in the virtual room across all conditions. All the animations generated were for one counselor/audience and retargeted to the other so that the audience behaviors were identical. Facial expressions were achieved using blendshapes on the high polygonal meshes. Facial expression for the counselor, the generic avatar, and the audience were designed to be positive (smiling). A real actor was used for creating the interaction with the virtual counselor; the actor read through the script (see Scripts below) and mocap data were recorded using the Glycon^[[5]](#footnote-5)^ instant motion capture software. For the follow-up session, where participants delivered the speech about Dire Straits on the stage in front of the virtual audience, the setup described previously^[[6]](#footnote-6),^^[[7]](#footnote-7)^ was used, but the participants were placed on the stage (Figure 2C) instead of in the audience to give their talk, and were then in the audience (Figure 2D).

## Scripts

### Conversation with counselor Single Exposure group

1. Hi, welcome to our session today. I am your counselor. Can you tell me a bit about yourself?
2. Great… OK, so the reason you are here today is because you become anxious when you must speak in front of an audience, like when you have to give a public speech. Can you tell me a bit more about that?
3. Hum, I see… I am glad that you’ve come to try to overcome your public speaking anxiety. It can be difficult to talk about your problem to someone you don’t know, but this is an important step in getting over it, and it’s great that you took it.
4. Let me tell you a bit about our session today. First, we will explore your anxiety a bit further. Then, I will explain to you how this is usually treated and how you can prepare for a public talk, and we will do a few exercises together. Do you agree with this?
5. Before we start, please tell me, on a scale from 0 to 10 how anxious do you feel right now, where 0 means you are totally relaxed and 10 you experience the highest anxiety and discomfort you have ever felt.
6. OK. So first, let’s explore further your anxiety problem. Do you have to give public speeches often?
7. What crowd do you usually need to give a speech to? Are they students, colleagues, or which others? Are they usually small or big groups?
8. Do you tend to avoid opportunities if they involve public speaking?
9. When was the last time you gave a public speech, for example, about your studies, your work or during a social event?
10. OK, great. Now let me tell you a few words about fear of public speaking and how we usually help people who suffer from it.
11. So, fear of public speaking is used to describe anxiety one feels whether in the situation of speaking in public or just thinking about the situation. Of course, it is typical for some people to feel anxious in such situations. Many people worry about whether the speech will go well, or about what other people will think, and then feel relieved when the speech is over. However, for some people, the anxiety may be so distressing that they avoid the situation altogether. While it is normal to avoid the things you fear, avoidance prevents you from learning that what you fear isn’t as dangerous as you think, and therefore doesn’t give you the chance to overcome it. So, to help people with fear of public speaking, one of the most effective psychological treatments is exposure therapy, where they gradually face their fears. Exposing oneself to the situation one fears, instead of avoiding it, allows you to get used to it: at first, people might feel quite anxious, but then, your body will start to relax, and the more you stay in that situation, the more comfortable you will feel. So, the next time you will have to be in that situation, you will feel less anxious, and so on.
12. Before we move on, please tell me, on a scale from 0 to 10 how anxious do you feel right now?
13. Great. Now that we have some context, let’s explore what happens to you when you speak in public. Can you think of a situation of public speaking in which you experienced anxiety, but were able to stay in the situation? If no situation comes to your mind, try to imagine what would happen in such a case and describe it to me.
14. Good, could you tell me what happened or what would happen to you in the period leading up to that situation?
15. Alright. And once you were in the situation, what were you thinking? For example, some people are concerned that the public might laugh, or that they won’t be interesting enough. What kind of thoughts came to your mind?
16. I see… and in your mind what was the worst thing that could happen in this situation?
17. And what would that lead to? What would be so bad about that?
18. What about your emotions? What emotions did you experience in this situation?
19. And how did your body feel? Did you notice any physical symptoms like trembling, sweating, blushing or other?
20. I see. And how do you react to these thoughts and feelings in that situation? For example, do you try to avoid looking at the public or do you tell yourself things to try to reassure yourself?
21. And what are the positive and negative consequences of this reaction?
22. OK. Thanks, that was very helpful. We now better understand the thoughts that come to your mind when you give a speech in public and the emotions and reactions that they trigger!
23. Very well then! Please tell me now, on a scale from 0 to 10 how anxious do you feel at this moment?
24. Alright. Now we’ll do a couple of activities that will allow you to gradually experience this anxiety relative to speaking in public. As I explained to you earlier, it is normal to experience some anxiety, but you are encouraged to stay as involved as possible. Don’t worry, we will start with something small, alright?
25. First, I would like you to count to 20 in your normal voice volume and at the speed you want.
26. Well done. Can you repeat the counting to 20 in a louder voice?
27. Great! And how anxious do you feel right now on a scale from 0 to 10?
28. Now I would like you to introduce yourself formally and share some information about you, such as your education background and what you currently do.
29. OK! How anxious do you feel right now?
30. Now, you can see on the projector screen a short monologue from the play HAMLET by William Shakespeare. I would like you to read it out loud.
31. OK. Great! Can you sing some part of a rhyme that you know or a favourite song? It does not need to be long.
32. Very Well! On a scale from 0 to 10 how anxious do you feel right now?
33. Nice! Now you’ll see a short movie on the screen, and I would like you to briefly describe what it is about.
34. Can you tell me if you have a favorite book or movie and briefly describe it? If you don’t have a favorite one, you can tell me about something that you read or watched recently. Please try to describe it in as much detail as possible.
35. Perfect! And how anxious do you feel right now?
36. We are almost at the end of our session and now I’d like to give you a little tip. A good way to feel confident when giving a planned speech is to prepare properly. Many people feel anxious before a speech because they don’t know what they are going to say, they haven’t prepared properly, and don’t know how to structure a talk. Does this ever happen to you?
37. OK, so a talk has a beginning – where you introduce the issues you’re going to talk about. Second, you divide the talk into different segments – for example, first I will talk about aspect X and then aspect Y, and so on. Third you go through these aspects one by one clearly marking the end of one and the beginning of the next. Finally, you return to the issues you raised at the start and say how each of these has been addressed. Then thank the audience and be ready to answer questions. Do you understand this general structure?
38. Very well then! For next time you will be given a task to do. You will have to give the welcome speech in front of a big audience for the opening of a concert performed by a famous band. I’ll share some material with you later. How does this sound?
39. So, to prepare you will need to do some research into how this is done taking into account the points we discussed before. It does not need to be a long speech and you will receive all the necessary information to prepare. If you have any questions, you can send them to me via email, alright?
40. Before you leave, please tell me on a scale from 0 to 10 how anxious this task makes you feel.
41. And how anxious do you feel right now in general?
42. I would now like to thank you for your time with me today and everybody here and to congratulate you for completing this session in front of everyone! You have done an excellent job! Take care and I will see you at the concert next week!

### Conversation with counselor Multiple Exposure group

The dialogue was identical to the Single exposure group but split over five sessions and small changes for the introduction and end of each. For example, session 2 was as follows:

1. Hello, how are you doing today?
2. It’s nice to see you again. Thanks for coming to our 2nd session. Before we start, do you feel anxious at all right now on a scale from 0 to 10, where 0 means you are totally relaxed and 10 the most anxious you’ve ever been?
3. Today we have three more people with us. Last time we talked a bit about your problem of anxiety in general and I explained how our therapy sessions work. Today let’s start by exploring what happens to you specifically when you speak in public. OK?
4. [Dialogue continues same as Single Session]
5. Alright. We’ll end our session here and next time you come we’ll do some exercises together, and we’ll also invite a couple more people to attend. Thank you and I’ll see you soon!

### Conversation Control condition group

1. Hello how are you! My name is X. Nice to meet you.
2. Where are you from?
3. I am from Y. Do you know the country? Have you ever been?
4. And what do you do for a living? I am a student at University of Z.
5. I see… And aside from this, do you have any hobbies? What do you like to do most in your free time?
6. Me…I love to play my guitar and read books.
7. And do you like movies?
8. Do you have any movies you've seen recently to recommend me?
9. Thanks, I haven't seen any for a long time.
10. So, do you like to travel? And what part of the trip do you like the most? I travel a lot and I like meeting new people.
11. Good! Do you have other brothers or sisters?
12. I have a younger sister. She goes to school.
13. And how was your day today? Have you done anything interesting?
14. Well, I'm pleased to meet you and I wish everything goes well for you! Take care! Goodbye!

1. [www.unity.com](file:///D:\Users\melslater\Documents\Research\ERC%202016\Research\Multiple%20Freud\Paper\Supplementary\www.unity.com) [↑](#footnote-ref-1)
2. [www.adobe.com/es/products/fuse.html](file:///D:\Users\melslater\Documents\Research\ERC%202016\Research\Multiple%20Freud\Paper\Supplementary\www.adobe.com\es\products\fuse.html) [↑](#footnote-ref-2)
3. [www.mixamo.com](http://www.mixamo.com) [↑](#footnote-ref-3)
4. Slater M, Neyret S, Johnston T, Iruretagoyena G, Alvarez de la Campa Crespo M, Alabèrnia-Segura M, et al. An experimental study of a virtual reality counselling paradigm using embodied self-dialogue. Scientific Reports. 2019 2019/07/29;9(1):10903. doi: 10.1038/s41598-019-46877-3. [↑](#footnote-ref-4)
5. <https://www.glycon3d.com/> [↑](#footnote-ref-5)
6. Beacco A, Oliva R, Cabreira C, Gallego J, Slater M. Disturbance and Plausibility in a Virtual Rock Concert: A Pilot Study. 2021 IEEE Virtual Reality and 3D User Interfaces (VR). 2021:538-45. doi: 10.1109/VR50410.2021.00078. [↑](#footnote-ref-6)
7. Slater M, Cabriera C, Senel G, Banakou D, Beacco A, Oliva R, et al. The sentiment of a virtual rock concert. Virtual Reality. 2022. doi: doi.org/10.1007/s10055-022-00685-9. [↑](#footnote-ref-7)
