# Supplementary material for: Desensitizing Anxiety Through Imperceptible Change: Feasibility Study on a Paradigm for Single-Session Exposure Therapy for Fear of Public Speaking
Source: JMIR Form Res. 2024 Jul 22;8:e52212. doi: 10.2196/52212 (PMC11301124; doi:10.2196/52212)
Supplement: Multimedia Appendix 4 [file formative_v8i1e52212_app4.docx]

# **Multimedia Appendix 4**

Table S2 shows the VR related questions administered after the VR exposure.

## Table S2. Post-VR experience Questionnaire

| **Variable** | **Question** |
| --- | --- |
| *Body* | To what extent did you feel that the virtual body you saw when looking down could be your own body (even if it did not look like you physically)?  1 = Not at all … 7 = Very much |
| *Mirror* | To what extent did you feel that the virtual body you saw when looking into the mirror could be your own (even if it did not look like you physically)?  1 = Not at all … 7 = Very much |
| *Agency* | To what extent did you feel that the movements of your virtual body were caused by your own movements?  1 = Not at all … 7 = Very much |
| *Feltaudience* | To what extent did you feel that there was an audience in front of you?  1 = Not at all … 7 = Very much |
| *Awareaudience* | How aware were you of having an audience in front of you?  1 = Not at all … 7 = Very much |
| *Impression* | What impression do you think you made on the audience?  1 = Very Negative … 7 = Very Positive |
| *Pleasant* | How nice/Pleasant was the audience?  1= not at all, 7 = very much |
| *Relaxed* | How comfortable and relaxed did you feel during the VR experience?  1= not at all, 7 = very much |
| *interaction* | How nice/pleasant was the interaction with the person you were talking to?  1= not at all, 7 = very much |
| *realperson* | To what extent did the person you spoke to act like a real person?  1= not at all, 7 = very much |
| *familiarity* | How familiar were you with the Dire Straits band before preparing your presentation?  1= not at all, 7 = very much |
| **Open-ended Questions** | |
| *essay1* | After completion of the first phase of the experiment (Single, Multiple, Control) the questionnaire included a final open-ended question:  “Please describe your experience in a few words (ideally at least 150 words) focusing on: your feeling to be at the depicted space and discussing with the virtual character, your conversation with the character and overall feelings/emotions that were generated; aspects that drew you into the experience; aspects that drew you out of the experience, and any other comments.” |
| *essay2* | After completion of the second phase of the experiment (introducing the concert) the questionnaire included a final open-ended question:  “Please add any other comments related to your experience (ideally at least 100 words) concentrating on your thoughts and feelings before starting the speech, while delivering the speech in front of the audience and after finishing the speech.” |
