# Supplementary material for: Desensitizing Anxiety Through Imperceptible Change: Feasibility Study on a Paradigm for Single-Session Exposure Therapy for Fear of Public Speaking
Source: JMIR Form Res. 2024 Jul 22;8:e52212. doi: 10.2196/52212 (PMC11301124; doi:10.2196/52212)
Supplement: Multimedia Appendix 5 [file formative_v8i1e52212_app5.docx]

# Multimedia Appendix 5 - Response Variables

## Personal report of communication apprehension (PRCA-24)

This instrument designed by [38] is the most widely used to help assess individuals’ personal level of communication apprehension. The instrument is designed to measure overall anxiety as well as anxiety in four specific communication contexts: interpersonal or dyadic, small group, meeting or large group, and public speaking. It is composed of 24 statements with 12 reverse items scored from 1 (Strongly Disagree) to 5 (Strongly Agree). Scores that are above the low (>51) and below the high (<80) indicate an average level of apprehension. It is highly reliable (Cronbach’s Alpha = .95) and has very high predictive validity. (Croucher et al. 2019) found that the PRCA-24 demonstrated strong reliability across different languages (i.e., French, German, Spanish, German, Kurdish, English, Russian) and national cultures (i.e., France, Germany, India, Iran, Kyrgyzstan, Rwanda, Spain, United Kingdom). The instrument was administered to participants before the first VR exposure *(prePRCA24)* and again after the VR treatment just before the follow-up session *(postPRCA24)*.

## State Perceived Index of Competence (SPIC)

The instrument developed by [39] was used to measure the state perceived communicative competence at three different times during the treatment sessions – at baseline *(preSPIC),* right before the follow-up session in relation to the speech they had to prepare *(postSPIC),* and right after the follow-up session *(afterSPIC)* in relation to the speech they presented in front of the audience*.* The scale comprises 7 positive items, indicating high competence, and 8 negative items, indicating low competence. The items are answered on a 7-point Likert scale ranging from 1 (strongly disagree) to 7 (strongly agree). Examples of positive and negative items given before the speech are "I am capable of giving a good speech today" and "I feel that I will mess-up this speech." The score ranges between 15 and 105 with a medium score of 60. It has shown high validity and reliability (Cronbach’s Alpha = .96).

## Short-form STAI

This measure [40] assessed state levels of anxiety prior to the follow-up session (public speaking task) related to how participants felt regarding the public speech they had to prepare for that day and while *anticipating* the speech (*postSTAI*). It is composed of items taken from the State-Trait Anxiety Inventory STAI.^[[1]](#footnote-1),^^[[2]](#footnote-2)^ The items were answered from on a 4-point Likert scale ranging from 1 (not at all) to 4 (very much so). Scores range between 8 and 32 with higher scores interpreted as less anxiety and more comfort.

## Post-VR experience questionnaire

The questionnaire (Multimedia Appendix 4, Table 4.1) includes questions related to body ownership and agency with respect to participants’ virtual body (variables *body, mirror, agency*), as well as other questions related to how participants evaluated the virtual audience (*feltaudience, awareaudience, impression, pleasant*) and overall conversation with the virtual character (counselor or generic avatar) (*relaxed*, *interaction, realperson*). Questions were asked on a 1 (‘not at all’) to 7 (‘very much’) scale. In the case of Single Exposure these questions were asked immediately after the session, and in the case of Multiple Exposure right after the end of the last exposure, while being asked to consider all previous exposures. They were also asked after the follow-up session and the presentation of the concert. A further question in preparation for their Dire Straits announcement asked before the announcement was their degree of familiarity with Dire Straits (*familiarity*). Note we did not require participants to know about Dire Straits, since in reality people often have to prepare talks about something unfamiliar and therefore need to do some research to find out more about it.

## Implicit Association Test (IAT)

Implicit fear of negative evaluation was measured using an adaptation of the IAT [37], that was developed to address personality traits such as self-esteem and self-concept^[[3]](#footnote-3)^. The test combines the task of categorizing pleasant versus unpleasant word meanings (e.g., accepted or rejected) and classifying items (e.g., me or they) into self and other categories. An IAT measure of implicit fear of negative evaluation is computed as the difference in mean categorization latency when self and liked attributes share the same response key (self + liked), as compared with self + rejected. The IAT effect thus measures how much easier it is for participants to categorize self-items with pleasant items (admired, popular, accepted, etc.) than self-items with unpleasant items (shunned, unwanted, disliked etc.). The IAT was administered to participants twice: right before their first or single virtual exposure (*preIAT*) and during the follow-up exposure (*postIAT*) before they have the public speech. Higher IAT scores are interpreted as less fear of negative evaluation as participants are faster associating self attributes with more pleasant/liked words (Self/Liked vs Other/Rejected) compared to the opposite pairings (Self/Rejected vs Others/Liked). It has been shown that IAT scores tend to show slightly stronger associations corresponding to the pairings of the combined block that is completed first^[[4]](#footnote-4)^, and so the order of the combined blocks was counterbalanced among participants^[[5]](#footnote-5)^. The IAT followed the standard procedures described in [37] and was administered using the Millisecond Inquisit^[[6]](#footnote-6)^ software.

1. Spielberger C. State-Trait Anxiety Inventory: Bibliography, 2nd Edn. Palo Alto. CA: Consulting Psychologists Press; 1989. [↑](#footnote-ref-1)
2. Spielberger CD, Gonzalez-Reigosa F, Martinez-Urrutia A, Natalicio LF, Natalicio DS. The state-trait anxiety inventory. Revista Interamericana de Psicologia/Interamerican Journal of Psychology. 1971;5(3 & 4). [↑](#footnote-ref-2)
3. Greenwald AG, Farnham SD. Using the implicit association test to measure self-esteem and self-concept. J Pers Soc Psychol. 2000 Dec;79(6):1022-38. PMID: 11138752. doi: 10.1037//0022-3514.79.6.1022. [↑](#footnote-ref-3)
4. Nosek BA, Greenwald AG, Banaji MR. Understanding and using the Implicit Association Test: II. Method variables and construct validity. Pers Soc Psychol Bull. 2005 Feb;31(2):166-80. PMID: 15619590. doi: 10.1177/0146167204271418. [↑](#footnote-ref-4)
5. Nosek BA, Greenwald AG, Banaji MR. The Implicit Association Test at age 7: A methodological and conceptual review. Automatic processes in social thinking and behavior. 2007:265-92. [↑](#footnote-ref-5)
6. <https://www.millisecond.com/> [↑](#footnote-ref-6)
