# Supplementary material for: Desensitizing Anxiety Through Imperceptible Change: Feasibility Study on a Paradigm for Single-Session Exposure Therapy for Fear of Public Speaking
Source: JMIR Form Res. 2024 Jul 22;8:e52212. doi: 10.2196/52212 (PMC11301124; doi:10.2196/52212)
Supplement: Multimedia Appendix 6 [file formative_v8i1e52212_app6.docx]

# Multimedia Appendix 6 – Participant Information by Condition

Table S3 shows the distribution of participants by condition, and their responses to a questionnaire administered at the first meeting.

Table S3. Participant Information by Condition.

| **Variable** | **Single** | **Multiple** | **Control** | **Overall** |
| --- | --- | --- | --- | --- |
| N | 16 | 14 | 15 | 45 |
| Age (Mean ± SD) | 29.0 ± 8.49 | 22.6 ± 4.38 | 26.3 ± 7.33 | 26.1 ± 7.36 |
| Gender  0 = male  1 = female  2 = other  3 = prefer not to answer | 8  8  0  0 | 2  10  1  1 | 4  9  2  0 | 14  27  3  1 |
| Education  0 = Less than high school degree  1 = High school degree or equivalent  2 = Some college but no degree  3 = Associate degree  4 = Bachelor degree  5 = Graduate degree | 0  3  9  4 | 0  1  10  3 | 1  3  4  7 | 1  7  23  14 |
| Computers: Median (IQR)  Expertise with computers: median (IQR)  1 = beginner…7 = expert | 4.5 (3) | 5(2) | 5(2) | 5(2) |
| Programming: Median (IQR)  Computer programming: median (IQR)  1 = Beginner…7 = Expert | 2.5 (4) | 2(3) | 2(4) | 2(4) |
| VR: Median (IQR)  Past VR experience  1 = Never…7 = Many times | 3(2.5) | 4(4) | 4(6) | 4(4) |
| Gamesyear: Median (IQR)  Median (IQR)  How many times have you played videogames (at home, at work, at school, in public places like shopping malls...) in the last year?  0 = 0  1 = 1-5  2 = 6-10  3 = 11-15  4 = 16-20  5 = 21-25  6 = >26 | 4(5) | 4(4) | 4(5) | 4(5) |
| Gamesweek: Median (IQR)  Video game playing over past week  0 = 0  1 = 1  2 = 2-3  3 = 3-5  4 = 5-7  5 = 7-9  6 = >10 | 1.5(3.5) | 1(5) | 2(3) | 1(3) |
