# Supplementary material for: Desensitizing Anxiety Through Imperceptible Change: Feasibility Study on a Paradigm for Single-Session Exposure Therapy for Fear of Public Speaking
Source: JMIR Form Res. 2024 Jul 22;8:e52212. doi: 10.2196/52212 (PMC11301124; doi:10.2196/52212)
Supplement: Multimedia Appendix 7 [file formative_v8i1e52212_app7.docx]

# Multimedia Appendix 7 – Results of the VR Questionnaire

Here we discuss the results of the questionnaire administered after the VR experience, relating to aspects of the VR itself.

## Body Ownership and Agency

On entering the VR participants had a life-sized virtual body that visually substituted their real body. If they would look down towards themselves, they would see this body instead of their real body. There was a mirror, and they would see the body reflected in the mirror (from the shoulders down, not including the head). The body moved according to their own upper body movements.

**Figure S1.** Box plots showing the median, interquartile range, and range by condition and time for the body ownership and agency questionnaire. Time refers to after the Conversation with the counselor or in the Control condition with the other person and after the follow-up when having given the Concert speech. The thick horizontal lines are the medians, the boxes are the interquartile ranges (IQR). The Whiskers range from max (min value, lower quartile – 1.5×IQR) to min (max value, upper quartile + 1.5×IQR). Values outside this range are shown individually.

Participants were administered the post-VR experience questionnaire after their VR exposure. In the case of Single Exposure these questions were asked immediately after the session, and in the case of Multiple Exposure right after the end of the last exposure, while being asked to consider all previous exposures. They were also asked after their presentation of the concert. Figure S1 shows the box plots for the body ownership and agency variables. Results are in line with previous results – all medians except for one are 5, with small interquartile ranges. The exception is *mirror* for Multiple Exposures, possibly because it was at the end and during the last session, participants concentrated on talking to the counselor and the audience and did not pay attention to the mirror. *Agency* is essentially a test of how well the tracking was working, as the movements of the avatar were caused by the movements of the participant.

## Response to the Audience

**Figure S2.** Box plots showing the median, interquartile range, and range by condition and time for participants’ responses to the audience. The thick horizontal lines are the medians, the boxes are the interquartile ranges (IQR). The Whiskers range from max (min value, lower quartile – 1.5×IQR) to min (max value, upper quartile + 1.5×IQR). Values outside this range are shown individually.

Figure S2 shows the box plots of responses to the audience or in the case of the Control Group the one person that participants were talking with (feltaudience, awareaudience, impression, pleasant) (from Multimedia Appendix 3). In all conditions the participants were aware of the audience and found it to be pleasant or neutral. Their view of the impression they made on the audience is moderate.

## Responses to the conversation and overall relaxation.

**Figure S3.** Box plots showing the median, interquartile range, and range by condition and time for participants’ reactions for the person they talked with or the concert scenario (audience). The thick horizontal lines are the medians, the boxes are the interquartile ranges (IQR). The Whiskers range from max (min value, lower quartile – 1.5×IQR) to min (max value, upper quartile + 1.5×IQR). Values outside this range are shown individually.

Figure S3 shows the box plots of responses related to the overall conversation with the virtual counselor or in the case of the Control Group the one that they talked with (relaxed, interaction, realperson) (Multimedia Appendix 3). The results show that for the Single and Multiple Exposures condition the counselor was evaluated as a real person and the interaction was pleasant.

## Familiarity with Dire Straits

A further question in preparation for their Dire Straits announcement asked before the announcement was participants’ degree of familiarity with Dire Straits (Multimedia Appendix 4). Figure S4 shows a lower level of familiarity for the Multiple condition compared with the other conditions.

**Figure S4.** Box plots showing the median, interquartile range, and range by condition regarding participants’ degree of familiarity with Dire Straits.
