# Supplementary material for: Desensitizing Anxiety Through Imperceptible Change: Feasibility Study on a Paradigm for Single-Session Exposure Therapy for Fear of Public Speaking
Source: JMIR Form Res. 2024 Jul 22;8:e52212. doi: 10.2196/52212 (PMC11301124; doi:10.2196/52212)
Supplement: Multimedia Appendix 8 [file formative_v8i1e52212_app8.docx]

# Multimedia Appendix 8 – Further Information on Results

## Posterior Distributions

Table S4 summarizes the posterior distributions of the parameters of the model. The mean and standard deviation columns show the means of the probability distributions. The credible intervals contain 95% of the distributions, with equal tails of 2.5%. The final column is the probability of the parameter being positive. If the mean of the distribution is positive then this can be considered as the probability that the corresponding variable is positively associated with the response. If the mean is negative then 1 – prob is the probability of the parameter being negative, i.e., the corresponding variable is negatively associated with the response. Probabilities of at least 0.8 are typically considered as noteworthy. It is important to note that all of these results are derived from the joint distribution of all the parameters, so there is no problem equivalent to multiple comparisons reducing ‘significance’ as is the case in null hypothesis significance testing.

**Table S4.** Summaries of the posterior distributions showing the means, standard deviations, and 95% equal interval credible intervals. Prob>0 is the posterior probability of the parameter being positive.

| **Parameter** | **Coefficient for** | **Mean** | **SD** | **2.5%** | **97.5%** | **Prob>0** |
| --- | --- | --- | --- | --- | --- | --- |
| *postIAT* |  |  |  |  |  |  |
| $\mu_{iat}$ |  | -0.46 | 0.38 | -1.20 | 0.29 | 0.111 |
| $\alpha_{iat,2}$ | Multiple | -0.38 | 0.60 | -1.58 | 0.79 | 0.256 |
| $\alpha_{iat,3}$ | Single | -0.66 | 0.59 | -1.84 | 0.48 | 0.130 |
| $\beta_{iat}$ | *preIAT* | -0.11 | 0.89 | -1.89 | 1.60 | 0.458 |
| $\gamma_{iat,2}$ | Multiple×*preIAT* | 1.44 | 1.64 | -1.75 | 4.70 | 0.817 |
| $\gamma_{iat,3}$ | Single×*preIAT* | 2.52 | 1.58 | -0.54 | 5.66 | 0.945 |
| $\phi_{iat}$ |  | 13.87 | 2.92 | 8.77 | 20.10 |  |
| ***postPRCA24*** |  |  |  |  |  |  |
| $\mu_{prc}$ |  | 12.10 | 7.83 | -3.20 | 27.46 | 0.942 |
| $\alpha_{prc,2}$ | Multiple | 28.47 | 13.90 | 1.34 | 55.83 | 0.979 |
| $\alpha_{prc,3}$ | Single | 19.67 | 11.63 | -3.66 | 42.60 | 0.953 |
| $\beta_{prc}$ | *prePRCA24* | 0.88 | 0.13 | 0.64 | 1.12 | 1.000 |
| $\gamma_{prc,2}$ | Multiple×*prePRCA24* | -0.37 | 0.18 | -0.74 | -0.01 | 0.023 |
| $\gamma_{prc,3}$ | Single×*prePRCA24* | -0.24 | 0.17 | -0.57 | 0.10 | 0.077 |
| $\nu_{prc}$ |  | 23.98 | 14.29 | 5.55 | 59.29 |  |
| $\sigma_{prc}$ |  | 8.53 | 1.09 | 6.69 | 11.00 |  |
| ***postSPIC*** |  |  |  |  |  |  |
| $\mu_{pspic}$ |  | 1.30 | 9.76 | -17.74 | 20.48 | 0.560 |
| $\alpha_{pspic,2}$ | Multiple | 47.78 | 14.46 | 18.74 | 75.80 | 1.000 |
| $\alpha_{pspic,3}$ | Single | 26.29 | 21.01 | -15.66 | 67.98 | 0.895 |
| $\beta_{pspic}$ | *preSPIC* | 0.83 | 0.14 | 0.55 | 1.10 | 1.000 |
| $\gamma_{pspic,2}$ | Multiple×*preSPIC* | -0.67 | 0.23 | -1.12 | -0.21 | 0.002 |
| $\gamma_{pspic,3}$ | Single×*preSPIC* | -0.33 | 0.31 | -0.93 | 0.29 | 0.142 |
| $\lambda_{pspic}$ | *familiarity* | 0.93 | 1.07 | -1.13 | 3.04 | 0.810 |
| $\nu_{pspic}$ |  | 22.28 | 14.03 | 4.84 | 57.32 |  |
| $\sigma_{pspic}$ |  | 10.50 | 1.38 | 8.11 | 13.44 |  |
| ***afterSPIC*** |  |  |  |  |  |  |
| $\mu_{aspic}$ |  | -9.68 | 12.57 | -34.58 | 15.25 | 0.211 |
| $\alpha_{aspic,2}$ | Multiple | 68.06 | 19.54 | 29.48 | 106.34 | 0.999 |
| $\alpha_{aspic,3}$ | Single | 51.42 | 26.65 | -2.07 | 103.07 | 0.968 |
| $\beta_{aspic}$ | *prePRCA24* | 0.84 | 0.18 | 0.47 | 1.19 | 1.000 |
| $\gamma_{aspic,2}$ | Multiple×*preSPIC* | -1.02 | 0.30 | -1.62 | -0.42 | 0.001 |
| $\gamma_{aspic,3}$ | Single×*preSPIC* | -0.52 | 0.39 | -1.28 | 0.28 | 0.096 |
| $\lambda_{aspic}$ | *familiarity* | 2.48 | 1.31 | -0.12 | 5.02 | 0.970 |
| $\nu_{aspic}$ |  | 23.48 | 14.58 | 4.99 | 61.43 |  |
| $\sigma_{aspic}$ |  | 13.20 | 1.73 | 10.23 | 17.02 |  |
| ***postSTAI*** |  |  |  |  |  |  |
| $\mu_{anx}$ |  | 17.85 | 1.93 | 14.03 | 21.66 | 1.000 |
| $\alpha_{anx,2}$ | Multiple | 1.63 | 1.89 | -2.14 | 5.32 | 0.812 |
| $\alpha_{anx,3}$ | Single | 2.43 | 1.67 | -0.86 | 5.69 | 0.927 |
| $\lambda_{anx}$ | *familiarity* | 0.73 | 0.41 | -0.06 | 1.53 | 0.965 |
| $\nu_{anx}$ |  | 25.37 | 14.34 | 6.75 | 61.27 |  |
| $\sigma_{anx}$ |  | 4.35 | 0.55 | 3.45 | 5.58 |  |

## Goodness of Fit of the Model

The statistical model provides a good fit to the data. This can be shown in two ways. If $y_{i}$ is any response variable for the $i$th individual then the model (Eq. 1) can be used to simulate new data on $y_{i}$called the ‘predicted posterior distribution’. Taking the mean of this distribution for each $i=1, 2, \ldots, n$ gives a point estimate for $y_{i}$. We can consider these as ‘fitted values’ of the model.

Table S5 shows the correlations between the means of the predicted posterior distributions and the observed values (and, solely as a measure of the fit, the 95% confidence intervals). The correlations are strong indicating a good fit between the predicted and observed values.

**Table S5.** Pearson Correlations and the 95% confidence intervals for the means of the predicted posterior distributions and the observed values.

| **Variable** | **Pearson Correlation** | **95% Confidence Interval** |
| --- | --- | --- |
| *postIAT* | 0.345 | 0.058 to 0.580 |
| *postPRCA24* | 0.888 | 0.804 to 0.937 |
| *preSPIC* | 0.746 | 0.579 to 0.853 |
| *postSPIC* | 0.770 | 0.615 to 0.867 |
| *postSTAI* | 0.381 | 0.099 to 0.607 |

The second method is called ‘leave-one-out’ cross validation^[[1]](#footnote-1)^. This involves, in principle, scarrying out the analysis using all data points but one and then estimating this data point from all the others, for each data point in turn. This results in a statistic referred to as the expected log pointwise predictive density (ELPD). There are corresponding ‘Pareto k estimates’ which, if high, reflect a lack of goodness of fit since the corresponding data value is not well predicted by the model (has high or infinite variance). The method also estimates the predicted number of parameters, which if high compared to the true values would indicate overfitting.

For each response variable the ELPD, Pareto k values, and the estimates number of parameters indicated no problems with the model fits. These results can be seen in the online data and programs.

## Data set and Software

The complete data and R code is available on

https://www.kaggle.com/code/melslater/multiple

This shows the execution of all R code.

To access the data and to interactively examine the code select the ‘Edit’ button at the top right-hand corner. This will give access to the actual results.csv file which contains the data.

You can then execute each cell in turn, by clicking the small arrow to its left hand side.

Alternatively, the data can be accessed directly on

https://www.kaggle.com/datasets/melslater/multiple-fops

Create a login on Kaggle to save the results if required.

1. Vehtari A, Gelman A, Gabry J. Practical Bayesian model evaluation using leave-one-out cross-validation and WAIC. Statistics and computing. 2017;27(5):1413-32. doi: 10.1007/s11222-016-9696-4. [↑](#footnote-ref-1)
