# Supplementary material for: Desensitizing Anxiety Through Imperceptible Change: Feasibility Study on a Paradigm for Single-Session Exposure Therapy for Fear of Public Speaking
Source: JMIR Form Res. 2024 Jul 22;8:e52212. doi: 10.2196/52212 (PMC11301124; doi:10.2196/52212)
Supplement: Multimedia Appendix 9 [file formative_v8i1e52212_app9.docx]

# Multimedia Appendix 9 – Sentiment Analysis

## Sentiment Analysis Background

Sentiment analysis^[[1]](#footnote-1),^^[[2]](#footnote-2)^ is a machine learning technique based on assignation of numerical scores to words in massive dictionaries. Each word is assigned a positive or negative valence. A score is derived for each piece of text, for example, as the average score over all the relevant words in the text, though modified by natural language analysis, for example, to take account of negations. Thus, sentiment analysis employs natural language processing, text analysis, and computational linguistics in order to give, for example, a greater score to ‘it was very good’ compared with ‘it was good’ and to understand that ‘it was not very good’ has negative sentiment, and thus a lower score than each of the previous two examples. Of course, it also deals with much more complex sentences than these.

There are many different sentiment analysis methods, Rinker^[[3]](#footnote-3),^^[[4]](#footnote-4)^ includes a comparative evaluation of several sentiment analysis packages^[[5]](#footnote-5),^^[[6]](#footnote-6)^. Our approach used 4 different sentiment analysis packages available through the R statistical programming language (https://www.r-project.org/about.html).^[[7]](#footnote-7),^^[[8]](#footnote-8)^ This was to capture different nuances of sentiment that vary between different approaches. We used sentimentr (https://github.com/trinker/sentimentr), the VADER system^[[9]](#footnote-9)^ with the R implementation^[[10]](#footnote-10)^ by Katherine Roehrick, the syuzhet package^[[11]](#footnote-11)^ (https://cran r-project org/web/packages/syuzhet), and SentimentAnalysis^[[12]](#footnote-12),^^[[13]](#footnote-13),^^[[14]](#footnote-14)^.

## Methods

After the finalisation of the first part of the study, where participants either had a single exposure or 5 multiple exposures, or in the control group, they were asked as part of the post-exposure questionnaire:

“Please describe your experience in a few words (ideally at least 150 words) focusing on: your feeling to be at the depicted space and discussing with the virtual character, your conversation with the character and overall feelings/emotions that were generated; aspects that drew you into the experience; aspects that drew you out of the experience, and any other comments.”

After the second part of the study, their presentation at the virtual concert, they were asked in the questionnaire:

“Please add any other comments related to your experience (ideally at least 100 words) concentrating on your thoughts and feelings before starting the speech, while delivering the speech in front of the audience and after finishing the speech.”

We refer to the texts written by the participants as ‘essays’. By applying each of the 4 sentiment methods mentioned above we obtained an $n\times4$ matrix, where $n$ is the number of texts and the 4 columns are the sentiment scores produced by the 4 packages. Then k-means cluster analysis (using kmeans in R) was used to find subsets of the texts that had similar scores. Given the sample sizes just two clusters were used for each of the two sets of texts (after the first and second phases), to avoid clusters with too few elements and to avoid overlap between the clusters.

A way to visualise the clusters is to find the principal components (PCs) of the 4 sentiment scores, and plot the scores per individual on the first two PCs. The R package factoextra^[[15]](#footnote-15),^^[[16]](#footnote-16),^^[[17]](#footnote-17)^ includes the function fviz_cluster that achieves this. We use the lexRank^[[18]](#footnote-18),^^[[19]](#footnote-19)^ method to summarise the text in the clusters. This produces a set of summary sentences ranked from highest to lowest salience. We reproduce the first 10 summaries.

## Results

### After exposure therapy (single or multiple sessions)

After the first phase (single exposure, multiple exposures, or control group) 39 participants wrote the short essays, with mean ± SD 102 ± 48.1 words, shown by condition in Table S6.

Figure S5 shows the two clusters, displayed on the first two principal components of the matrix that contains all 4 cluster scores. It is clear that the two clusters are well separated on the first principal component, but not on the second.

Table S7 shows the lexRank summary sentences. It is clear that cluster 2 represents more positive sentiment.

**Table S6**. Means and SDs of the number of words in the essays by condition after exposure therapy

| **Condition** | **Mean** | **SD** | **n** |
| --- | --- | --- | --- |
| **Single** | 51.4 | 16 | 16 |
| **Multiple** | 116.0 | 48.9 | 14 |
| **Control** | 72.6 | 27.9 | 9 |


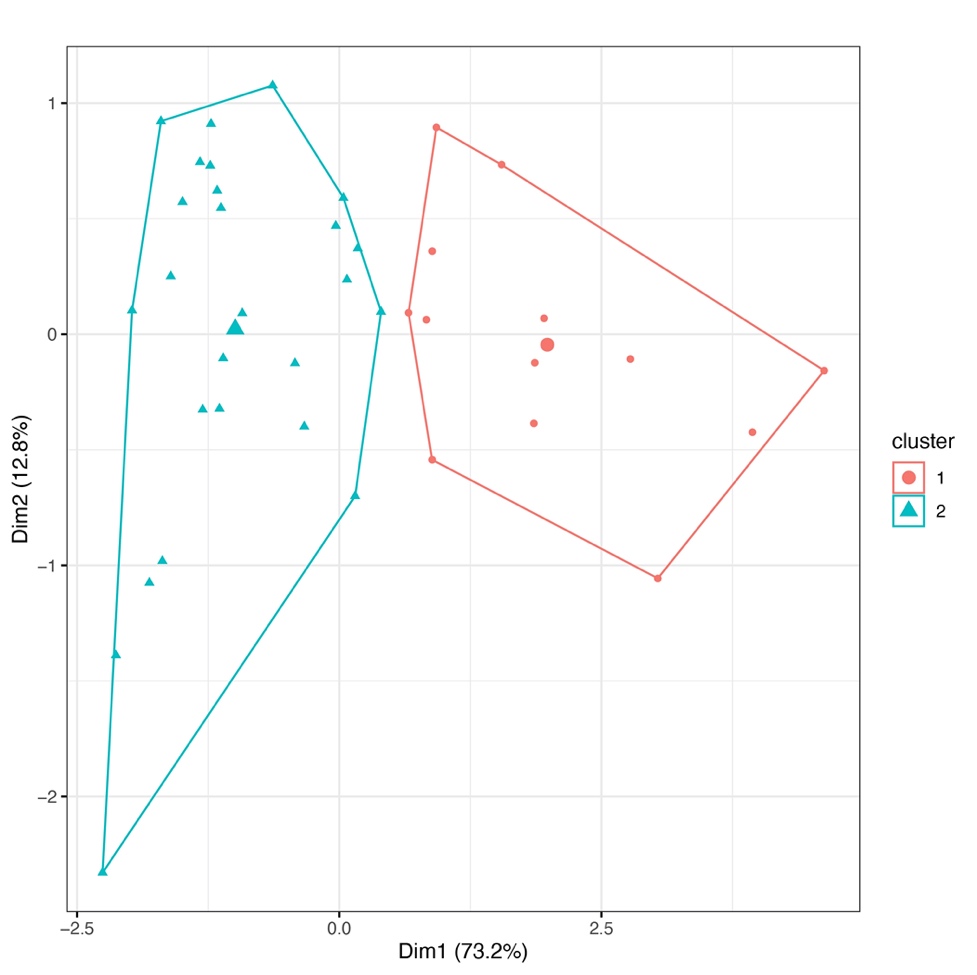


**Figure S5.** Plot of the first two principal components (Dim1 and Dim2) of the 39×4 matrix of sentiment scores over all essays after the exposure therapy, with the clusters shown by the convex hulls of their corresponding points. Cluster 1 contains 13 points and cluster 2 contains 26 points.

**Table S7**. Summaries of the two clusters after the exposure therapy based on the lexRank method.

| **Cluster 1** | **Cluster 2** |
| --- | --- |
| 1 . I have felt inside the experience at all times, and I have had the feeling that I was talking to a real person. | Regarding the virtual character, the experience was really good, I was feeling that he was like a friend helping me. |
| 2. I was a little nervous and uncomfortable when I started talking to the virtual character, since deep down I was aware that he was not a real person and that made me feel a little different. | In general, it has been a completely new experience in which interacting with the virtual character has been like talking to a real person. |
| 3. When I was talking to a virtual character, I was talking as if there was a real person in front of me. | I have felt very immersed in the environment created and at no time have I felt out of the experience. |
| 4.It seemed to me a quite realistic experience, where the person with whom I was speaking gave me the impression that he understood what I felt and that is why he made me do tasks that were increasingly difficult for me. | My feeling in this experience was quite new, being in a virtual environment and not knowing what I would have to expose myself to, but once the tasks or conversations were happening, my feeling of anxiety for not knowing what I had to do started decreasing. |
| 5. Coming out of the experience it was as if I woke up suddenly, but it didn't bother me at all and I didn't feel bad at any time, just uncomfortable with the situation and a little anxious during the conversation. | I think it has been a very good experience and I have felt inside the room with a virtual audience, the conversation was very pleasant with the person who was in front of me and although it is true that I have noticed that characters came out of the character who was in front of me, in the end I didn't realize there were so many. |
| 6. I could see that the person I was talking to was changing and becoming the public. | Perhaps one aspect that took me out of the experience is having the character you interact with so close because you feel that he is too much on top of you maybe and the audience was more in the background. |
| 7. Although at first I was puzzled that the audience came from the same person, in the end I didn't pay much attention and I wasn't so nervous. | What attracted me to this experience was being able to improve public speaking. |
| 8. I didn't like having to speak to them and felt like I didn't have anything interesting to say. | As for the rest of the experience, I felt quite inside. |
| 9. It was strange the fact of talking to a virtuous person, causing him to interfere in the conversation, since it made me a little nervous and I didn't converse much with him. | The experience in general has been great, I liked it a lot and both the exercises and the conversation that I have had with my interlocutor made me feel very comfortable and attracted me to live this experience. |
| 10. But when the woman appeared in front of me, I wasn't sure if she had to answer out loud and that made me feel quite uncomfortable. | I felt attracted to the experience of being able to treat something that can be traumatic on certain occasions in a virtual way and I thought it would be an ideal way to overcome those fears. |

We can also consider the distribution of condition by cluster, as in Table S8. This shows that - for this sample - there is a difference between the single and multiple exposure sessions (i.e., those that had the exposure therapy) and the control. Those that had the exposure therapy were more than twice as likely to be in the high sentiment cluster than the low sentiment one, whereas the control group were almost equal between the two clusters.

**Table S8.** Distribution of condition by cluster after the exposure therapy

|  | **Cluster 1** | **Cluster 2** |
| --- | --- | --- |
| **Single** | 5 | 11 |
| **Multiple** | 3 | 11 |
| **Control** | 5 | 4 |

## After the concert presentation

In this case all 45 of the participants wrote the short essays, with mean number of words 93 ± 56.3.

**Table S9**. Means and SDs of the number of words in the essays by condition after introducing the concert

| **Condition** | **Mean** | **SD** | **n** |
| --- | --- | --- | --- |
| Single | 99.4 | 63.6 | 16 |
| Multiple | 109.0 | 52.1 | 14 |
| Control | 71.5 | 48.1 | 15 |

Figure S6 shows the two clusters, well separated on the first principal component, and from Table S10 it is clear that cluster 2 has greater sentiment than cluster 1.


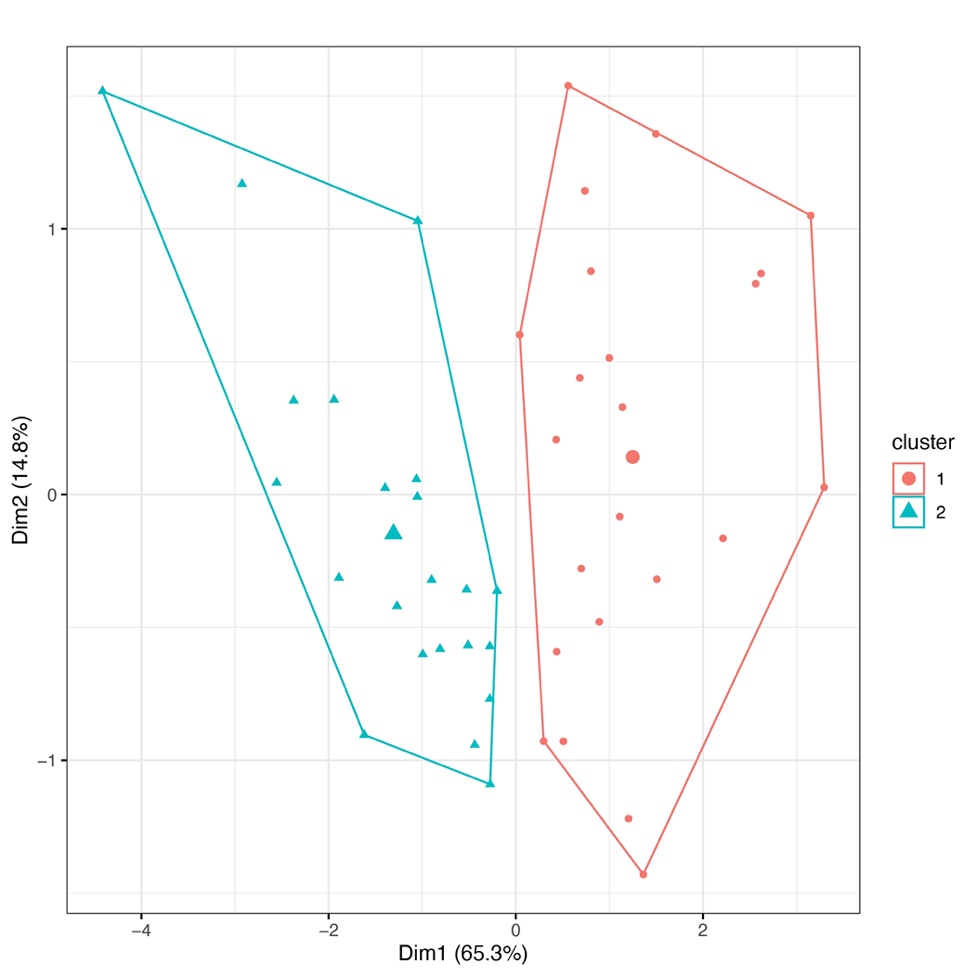


**Figure S6.** Plot of the first two principal components (Dim1 and Dim2) of the 39×4 matrix of sentiment scores over all essays after the concert presentation, with the clusters shown by the convex hulls of their corresponding points. Cluster 1 contains 23 points and cluster 2 contains 22 points.

**Table S10.** Summaries of the two clusters after the concert presentation based on the lexRank method.

| **Cluster 1** | **Cluster 2** |
| --- | --- |
| 1. The public was talking and I didn't know if I should start presenting myself. | After finishing the speech I felt relaxed and was able to enjoy the concert. |
| 2. Before starting the talk I felt very tense and nervous, because I knew it was not going to go well. | After giving the speech and being part of the audience I was able to relax and take in my surroundings. |
| 3. Before starting the speech, I felt like I was the person who gave a speech in front of the audience. | Before giving the speech I felt a bit nervous because I wanted to make a good impression and at the same time not forget any important information for my speech. |
| 4. From the beginning I felt very nervous because I thought I would go blank and forget important things, the moment alone with me repeating the talk before starting has helped me to put it in order and see how it would come to me without having the opportunity to read, after having finished it I went to do it in front of the public and I waited a few seconds for silence, but it didn't, so I started to get very nervous and I said the whole speech as I had planned. | During the speech I felt like I might forget what I was planning to say but I was able to remember enough to keep going until the end. |
| 5. Well, before the talk I felt some nervousness but I started to think that being more virtual and such was not going to affect me, but later when I started it I felt somewhat overwhelmed, and I kept feeling nervous and babbling... | Before the speech I was nervous because I did not think I knew enough information to make a good speech. |
| 6. I think that the experience of speaking to an audience is quite similar to what it would be in the real world, the audience was looking attentively as if they were human but I think that when I finished the speech when I was in the crowd this effect failed more than on stage since several people in the public were looking at you in addition to the concert which is a strange situation that would not happen in the real world. | I really enjoyed giving the speech, even if I forgot some parts. |
| 7. However, once I found myself in front of the large audience, it felt more real than expected. | It made me feel more relaxed and more able to have fun with the speech rather than taking it too seriously. |
| 8. It felt like being on a real stage talking to real people, however if it were real life i feel i would have a bit different reaction from the audience, like more interaction. | Before starting the speech I was quite nervous. |
| 9. I was really nervous before giving the talk and just wanted it to be over. | I felt like the speech didn't matter as much after the band came on, which is what everyone was waiting for anyway, and they all just wanted to have fun, not criticize my speech. |
| 10. In the end, during the talk it went fatal, I didn't follow the presentation I had planned at all because the words didn't come out, and I didn't remember the name of the single they released and I had to avoid saying it, when just taking off the equipment made me has come to mind as it was said, just like the surnames of the people who made up the band. | The speech was not very well spoken or informative but I hope it expressed my desire to get the audience excited about the band. |

Table S11 shows the distribution of condition by cluster. In this case, and for this sample, those in the Single condition were more than twice as likely to express higher sentiment, whereas those in the Multiple condition were roughly evenly spread across the two clusters, and those in the Control condition were twice as likely to express lower sentiment.

**Table S11.** Distribution of condition by cluster for essays after the concert

|  | **Cluster 1** | **Cluster 2** |
| --- | --- | --- |
| **Single** | 5 | 11 |
| **Multiple** | 8 | 6 |
| **Control** | 10 | 5 |

## Data set and Software for the Sentiment Analysis

The complete data and R code is available on

https://www.kaggle.com/code/melslater/sentiment-for-fops

This shows the execution of all R code and gives access to the data.

Create a login on Kaggle to save the results if required.

1. Bakshi RK, Kaur N, Kaur R, Kaur G, editors. Opinion mining and sentiment analysis. 2016 3rd International Conference on Computing for Sustainable Global Development (INDIACom); 2016: IEEE. [↑](#footnote-ref-1)
2. Liu B. Sentiment analysis and opinion mining. Synthesis lectures on human language technologies. 2012;5(1):1-167. [↑](#footnote-ref-2)
3. <https://github.com/trinker/sentimentr#comparing-sentimentr-syuzhet-meanr-and-stanford> [↑](#footnote-ref-3)
4. https://github.com/trinker/sentimentr [↑](#footnote-ref-4)
5. Yoon S, Parsons F, Sundquist K, Julian J, Schwartz JE, Burg MM, et al. Comparison of Different Algorithms for Sentiment Analysis: Psychological Stress Notes. Stud Health Technol Inform. 2017;245:1292. PMID: 29295377. [↑](#footnote-ref-5)
6. Naldi M. A review of sentiment computation methods with R packages. arXiv preprint arXiv:190108319. 2019. [↑](#footnote-ref-6)
7. Beacco A, Oliva R, Cabreira C, Gallego J, Slater M. Disturbance and Plausibility in a Virtual Rock Concert: A Pilot Study. 2021 IEEE Virtual Reality and 3D User Interfaces (VR). 2021:538-45. doi: 10.1109/VR50410.2021.00078. [↑](#footnote-ref-7)
8. Slater M, Cabriera C, Senel G, Banakou D, Beacco A, Oliva R, et al. The sentiment of a virtual rock concert. Virtual Reality. 2022. doi: doi.org/10.1007/s10055-022-00685-9. [↑](#footnote-ref-8)
9. Hutto C, Gilbert E, editors. Vader: A parsimonious rule-based model for sentiment analysis of social media text. Proceedings of the International AAAI Conference on Web and Social Media; 2014. [↑](#footnote-ref-9)
10. <https://CRAN.R-project.org/package=vader> [↑](#footnote-ref-10)
11. <https://github.com/mjockers/syuzhet> [↑](#footnote-ref-11)
12. <https://CRAN.R-project.org/package=SentimentAnalysis> [↑](#footnote-ref-12)
13. <https://mran.microsoft.com/snapshot/2017-08-06/web/packages/SentimentAnalysis/vignettes/SentimentAnalysis.html> [↑](#footnote-ref-13)
14. Feuerriegel S, Proellochs N, Feuerriegel MS. Package ‘SentimentAnalysis’. CRAN: London, UK. 2018 [↑](#footnote-ref-14)
15. <https://cran.r-project.org/web/packages/factoextra/index.html> [↑](#footnote-ref-15)
16. Kassambara A, Mundt F. Package ‘factoextra’. Extract and visualize the results of multivariate data analyses. 2017;76. [↑](#footnote-ref-16)
17. Kassambara A. Practical guide to principal component methods in R: PCA, M (CA), FAMD, MFA, HCPC, factoextra: Sthda; 2017. ISBN: 1975721136. [↑](#footnote-ref-17)
18. <https://www.rdocumentation.org/packages/lexRankr/versions/0.5.2/topics/lexRank> [↑](#footnote-ref-18)
19. Erkan G, Radev DR. Lexrank: Graph-based lexical centrality as salience in text summarization. Journal of artificial intelligence research. 2004;22:457-79. [↑](#footnote-ref-19)
